# Feilike and Its Constituent Licochalcone B Trigger Caspase-3/GSDME-Mediated Pyroptosis in Triple-Negative Breast Cancer via Modulation of the Mutant p53–Calcium/ER Stress–ROS–MAPK Axis

**DOI:** 10.3390/antiox15050649

**Published:** 2026-05-21

**Authors:** Jue Yang, Peng Zhao, Lianghong Zhou, Hui Song, Zili Feng, Hongjian Cui, Yanmei Li, Jianfei Qiu, Xiaojiang Hao

**Affiliations:** 1 State Key Laboratory of Discovery and Utilization of Functional Components in Traditional Chinese Medicine, Natural Products Research Center of Guizhou Province, Guizhou Medical University, Guiyang 550014, China; yangjue@gmc.edu.cn (J.Y.); zhaopeng960528@163.com (P.Z.); zhoulianghong1996@163.com (L.Z.); fengzili0328@163.com (Z.F.); cuihongjian3716@163.com (H.C.); liyanmei518@hotmail.com (Y.L.); 2 Department of Medical Laboratory, Guizhou Provincial Staff Hospital, Guiyang 550025, China; 3 School of Pharmaceutical Sciences, Guizhou Medical University, Guiyang 561113, China; 4 Key Laboratory of Endemic and Ethnic Diseases, Ministry of Education, Guizhou Medical University, Guiyang 561113, China; songhui620@gmc.edu.cn; 5 State Key Laboratory of Phytochemistry Kunming Institute of Botany, Chinese Academy of Sciences, Kunming 650201, China; 6 Yunnan Characteristic Plant Extraction Laboratory, Kunming 650201, China

**Keywords:** Feilike, pyroptosis, ROS, oxidative stress, endoplasmic reticulum stress, triple-negative breast cancer

## Abstract

Triple-negative breast cancer (TNBC) is an aggressive subtype of breast cancer with limited targeted therapeutic options, underscoring the urgent need for novel treatment strategies. Feilike (FLK), a Traditional Chinese Medicine formula with heat-clearing and detoxifying properties, aligns with key pathological features implicated in breast cancer progression. In addition, several of its components have demonstrated anti-tumor activity, positioning FLK as a potential therapeutic candidate for TNBC. In this study, we employed an integrated approach combining network pharmacology, transcriptomic analysis, and experimental validation to investigate the anti-TNBC effects of FLK. Our results demonstrate that FLK significantly inhibits the proliferation of TNBC cell lines and patient-derived organoids and induces typical pyroptotic features, including cell swelling and increased lactate dehydrogenase (LDH) release. Mechanistically, FLK triggers a mutant p53 signaling cascade involving calcium dysregulation, endoplasmic reticulum stress (ERS) activation, mitochondrial dysfunction, and reactive oxygen species (ROS) accumulation, which collectively activate the P38/JNK–Caspase-3/GSDME pathway to induce pyroptosis. In vivo, FLK markedly suppresses tumor growth in a 4T1 orthotopic mouse model and enhances the anti-tumor efficacy of Cyclophosphamide. Furthermore, Licochalcone B (LCB) is identified as a key bioactive constituent that recapitulates the pyroptosis-inducing effects of FLK. Collectively, our findings uncover a previously unrecognized mutant p53–ERS–ROS–MAPK signaling axis underlying FLK-induced pyroptosis and provide mechanistic insight and experimental evidence supporting the repurposing of FLK as a potential therapeutic strategy for TNBC.

## 1. Introduction

Breast cancer is a malignant tumor characterized by the uncontrolled proliferation of epithelial cells. According to the global cancer statistics 2022, breast cancer has become the primary factor endangering female health, with an incidence of 23.8% and a mortality rate of 15.4% [1]. Based on molecular characteristics, breast cancer can be classified into three types, including hormone receptor-positive (HR), human epidermal growth factor receptor 2-positive (HER-2) and triple-negative breast cancer (TNBC) [2]. Due to the lack of actionable molecular targets, TNBC patients are difficult to benefit from endocrine therapy and HER-2-targeted therapy [3]. Therefore, chemotherapy remains the primary treatment option for TNBC [3,4]. However, the clinical efficacy of chemotherapy is often limited by low selectivity, systemic toxicity and drug resistance [5,6]. These limitations highlight the urgent need to develop novel therapeutic strategies for TNBC.

Traditional Chinese Medicine (TCM) is known for its multi-component and multi-target therapeutic properties and has been extensively applied in cancer treatment. Previous studies have demonstrated that several TCM formulas with heat-clearing and detoxifying properties confer anti-breast cancer activity, such as Jin’gan capsules, San Ying decoction, Yiai Fuzheng formula and Xi Huang pill [7,8,9,10]. Feilike (FLK), a clinically approved TCM formula composed of multiple herbal medicines, is traditionally used for respiratory diseases and exhibits heat-clearing, detoxifying, and phlegm-resolving effects. Notably, some of its constituent herbs, such as *Scutellaria baicalensis* Georgi and *Scleromitrion diffusum* (Willd.) R. J. Wang have been reported to possess anti-TNBC activity [11,12]. These findings suggest that FLK may exert potential therapeutic effects against TNBC; however, its anti-tumor efficacy and underlying mechanisms remain largely unclear.

Conventional chemotherapeutic agents primarily eliminate tumor cells by inducing apoptosis [13]. However, sustained treatment often leads to acquired resistance due to apoptosis evasion [14]. Pyroptosis is a gasdermin-mediated programmed cell death characterized by membrane pore formation and release of pro-inflammatory cytokines such as IL-1β and IL-18, which potently activate anti-tumor immune responses [15,16]. Accumulating evidence suggests that pyroptosis induction represents a promising therapeutic strategy for TNBC. A variety of compounds, including hypericin [17], tetraarsenic hexoxide [18], nigericin [19], shikonin [20], curcumin [21], and baicalin [22], have been shown to suppress TNBC progression or enhance anti-tumor immunity through pyroptosis-mediated mechanisms.

Despite these advances, studies investigating clinically approved TCM formulas as pyroptosis inducers remain limited. Given their established safety profiles and clinical applicability, repurposing such formulas may provide an efficient and translational therapeutic strategy. In this study, we aimed to systematically investigate the anti-TNBC effects of FLK and elucidate its underlying mechanisms. Using TNBC cell lines, patient-derived organoids, and an orthotopic mouse model, we evaluated the anti-TNBC potential of FLK. In parallel, we employed ultra-high-performance liquid chromatography–high-resolution tandem mass spectrometry (UPLC-HR-MS/MS), network pharmacology, transcriptomic analysis, and experimental validation to identify its active constituents and elucidate the signaling pathways involved.

## 2. Materials and Methods

### 2.1. Preparation of FLK Extract and Chemicals

FLK was purchased from Guizhou Jianxing Pharmaceutical Co., Ltd. (Guiyang, China) and prepared in accordance with the method described in a previous patent (CN201910165098.9). The formulation consisted of the following crude herbs: *Scutellaria baicalensis* Georgi (173 g), *Peucedanum praeruptorum* Dunn (167 g), *Stemona sessilifolia* (Miq.) Miq (160 g), *Gentiana rhodantha* Franch (150 g), *Firmiana plantanifolia* (L.f.) Marsili (130 g), *Aster ageratoides* Turcz (96 g), and *Scleromitrion diffusum* (Willd.) R. J. Wang (120 g). *Scutellaria baicalensis* Georgi was pulverized and passed through a 16 mm sieve. A portion of *Stemona sessilifolia* (Miq.) Miq was further ground into a fine powder (<80 mesh) and reserved. The remaining *Stemona sessilifolia* (Miq.) Miq was combined with the other six herbs and subjected to two rounds of aqueous decoction. The first decoction was performed using eightfold (*w*/*v*) distilled water, followed by a second decoction using sixfold (*w*/*v*) water for 1.5 h. The combined extracts were filtered and concentrated to obtain a concentrated extract. The concentrated solution was then freeze-dried to obtain the FLK freeze-dried powder. Licochalcone B (LCB) and Cyclophosphamide (CTX) were obtained from Solarbio (Beijing, China) and Baxter Oncology Gmbh (Halle, Germany), respectively.

### 2.2. Cell Culture

The TNBC cell lines MDA-MB-231, BT-549 and 4T1 were acquired from ATCC (Manassas, VA, USA). MDA-MB-231 and 4T1 cells were maintained in DMEM (Gibco, Grand Island, NY, USA) supplemented with 10% FBS (Gibco, Grand Island, NY, USA) and 1% penicillin-streptomycin (Hyclone, Logan, UT, USA). BT-549 cells were cultured in RPMI 1640 medium (Gibco, Grand Island, NY, USA) containing 10% FBS, 0.1% insulin (Procell, Wuhan, China), and 1% penicillin-streptomycin. All cell lines were maintained at 37 °C in a CO_2_-humidified atmosphere.

### 2.3. Cell Viability Assay

The 3-(4,5-dimethylthiazol-2-yl)-2,5-diphenyltetrazolium bromide (MTT) assay was employed to assess cell viability. Cells (6 × 10^3^ cells/well) were seeded in 96-well plates. After cell adhesion, the cells were treated with various concentrations of FLK or LCB in each well for 24–72 h. Then, the 10 μL of MTT solution (Solarbio, Beijing, China) was added to each well and incubated for 4 h at 37 °C to form purple formazan crystals. Afterward, 100 μL of dimethyl sulfoxide (DMSO) solution (Solarbio, Beijing, China) was added to each well to dissolve the purple formazan crystals. Then, the absorbance at 490 nm was measured.

### 2.4. Colony Formation Assay

The colony formation assay was performed to evaluate cell proliferative ability. Cells (1000 cells/well) treated with FLK were plated in 6-well plates. After 14 days, the cells were washed twice with phosphate-buffered solution (PBS) (Solarbio, Beijing, China) and fixed with methanol (Aladdin, Shanghai, China) for 30 min. Next, the cell colonies were stained with crystal violet staining (Solarbio, Beijing, China) in the dark for 15 min. After being washed three times with PBS, the cell colonies were counted, and each blue-stained spot was defined as a single colony.

### 2.5. Organoid Culture

Fresh clinical TNBC tissue specimens were preserved in tissue activity protection solution (Orgen Biotechnology, Guangzhou, China) at 4 °C for ≤72 h. Under sterile conditions, extraneous (fat and muscle) and necrotic tissues were removed; the tissue was rinsed with Buffer P1 (Orgen Biotechnology, Guangzhou, China), minced into 1–2 mm fragments, and digested with Solution B (Orgen Biotechnology, Guangzhou, China) at 37 °C for 1–2 h, with vortexing every 30 min. DMEM/F12 (Orgen Biotechnology, Guangzhou, China) was added to stop digestion upon flocculation, followed by pipetting to prepare a cell suspension. The suspension was filtered through a 100 μm strainer (Orgen Biotechnology, Guangzhou, China), centrifuged at 300× *g* for 5 min, and subjected to erythrocyte lysis buffer (Orgen Biotechnology, Guangzhou, China). Isolated cells were resuspended in breast cancer organoid medium (Orgen Biotechnology, Guangzhou, China), mixed with growth factor-reduced Matrigel (Orgen Biotechnology, Guangzhou, China) at 1:1.5, seeded into non-tissue culture-treated 24-well plates, and solidified at 37 °C. Cultures were maintained at 37 °C with 5% CO_2_, and the medium was replenished every other day. All clinical TNBC tissue samples were collected with the approval of the Human Research Ethics Committee of Guizhou Medical University (Approval Number: 2024-105).

### 2.6. Organoid Viability

TNBC organoids were seeded at a density of 50–100 organoids per well. After 24 h, the organoids were treated with FLK (1 mg/mL) or LCB (40 μM). Following 24 h of treatment, viability was measured using a Cell Counting Kit-8 (CCK-8) (Mutual Success Technology, Guangzhou, China) assay, with absorbance read at 450 nm after 2 h of incubation. In parallel, a separate set of treated organoids was stained using a Live/Dead assay kit (Uelandy, Suzhou, China) according to the manufacturer’s instructions. Fluorescence images were captured, with live cells visualized by Calcein-AM (494/517 nm) and dead cells by propidium iodide (PI, 528/617 nm).

### 2.7. Lactate Dehydrogenase (LDH) Detection

The LDH detection was applied to evaluate the integrity of the cell membrane. Cells were seeded in 24-well plates (3 × 10^4^ cells/well). After overnight incubation, the cells were treated with indicated concentrations of FLK for 24 h. The supernatant was collected as the extracellular LDH content. Cells were then incubated with the LDH release reagent in a 37 °C incubator. The supernatant was collected as the intracellular LDH content. All supernatants were transferred to 96-well plates and 60 μL of LDH working solution (Beyotime, Shanghai, China) was added to each well. The absorbance was measured at 490 nm.

### 2.8. Propidium Iodide (PI) Staining Assay

The PI staining assay was used to detect dead cells. The cells were plated in 6-well plates (3 × 10^5^ cells/well) and treated with serial dilutions of FLK or LCB for 24 h. Then, the cells were stained with PI staining (BD Biosciences, Franklin Lakes, NJ, USA) in the dark for 15 min. Fluorescence images were captured by a fluorescence microscope. Images were quantitatively analyzed using ImageJ software (Version 1.54f, Wayne Rasband and contributors National Institutes of Health, Bethesda, MD, USA).

### 2.9. UPLC-HR-MS/MS Analysis

The chemical constituents of the FLK extract were identified using UPLC-HR-MS/MS on an Agilent 1100 system and a Thermo Ultimate 3000/Q EXACTIVE FOCUS mass spectrometer (Thermo Fisher Scientific, San Jose, CA, USA). Chromatographic separation was achieved on an ACE Ultracore 2.5 SuperC18 column (2.1 mm × 100 mm) using gradient elution with acetonitrile (A) and water (B) at a flow rate of 0.2 mL/min. The column temperature was maintained at 40 °C, and the injection volume was 5 μL. Mass spectrometry analysis was performed using an electrospray ionization (ESI) source operating in both positive and negative ion full-scan modes, with a mass scan range of *m*/*z* 100–1500 and a resolution of 70,000. The capillary temperature was set at 320 °C, the heater temperature at 350 °C, and the sheath gas and auxiliary gas flow rates were 35 and 10 arb, respectively. The acquired MS/MS fragmentation spectra were matched against the mzCloud online database and the Orbitrap Traditional Chinese Medicine Library (OTCML) for compound identification.

### 2.10. Network Pharmacology Analysis

According to oral bioavailability (OB) > 30% and drug-likeness (DL) > 0.18 or high gastrointestinal absorption, with at least two YES for DL, the target genes of active ingredients were collected in the TCMSP and SwissADME databases. TNBC-related genes were identified via the GeneCards, OMIM, PharmGkb, TTD and DrugBank databases. After identifying the intersection genes, Gene Ontology (GO) and Kyoto Encyclopedia of Genes and Genomes (KEGG) analyses were conducted on the Metascape platform. In addition, Cytoscape was used to construct the FLK-compound-target network. Finally, the protein–protein interaction (PPI) network was generated using the STRING database and further optimized by Cytoscape.

### 2.11. RNA Sequencing (RNA-Seq) Analysis

RNA-seq analysis was performed to analyze differential expression genes (DEGs) via the BGI platform (BGI, Shenzhen, China). In brief, after 24 h of treatment with FLK, total RNA was extracted via the TRIzol reagent (Invitrogen, Carlsbad, CA, USA). Then, according to the criterion of |log2 fold change| > 1 and *p* < 0.05, the DEGs were identified. GO and KEGG analyses were conducted on the Metascape platform.

### 2.12. Measurement of Reactive Oxygen Species (ROS)

The level of ROS was measured according to the manufacturer’s procedures of the ROS assay kit (Beyotime, Shanghai, China). The cells (3.0 × 10^5^), treated with FLK, were washed twice with PBS and incubated with DCFH-DA for 20 min in the dark. Subsequently, fluorescent images were captured by a fluorescence microscope and DCF fluorescence intensity was analyzed via flow cytometry (BD Biosciences, San Jose, CA, USA), with precisely 10,000 cellular events analyzed per sample.

### 2.13. Measurement of Mitochondrial Membrane Potential (MMP)

The MMP was detected by the JC-1 assay kit (Solarbio, Beijing, China). The cells were seeded in 6-well plates (3.0 × 10^5^ cells/well) and incubated with serial dilutions of FLK for 24 h. After washing twice with PBS, the cells were cultured with the JC-1 working solution at 37 °C for 20 min. Then, the cells were washed twice with the JC-1 buffer solution and the changes in red/green fluorescence were observed under a fluorescence microscope (Leica Microsystems, Wetzlar, Germany). Next, the cells were harvested and the fluorescence intensity was measured by flow cytometry (BD Biosciences, San Jose, CA, USA), with exactly 10,000 cellular events analyzed per sample.

### 2.14. Measurement of Ca^2+^

The content of Ca^2+^ was examined through the Fluo-4 AM (Beyotime, Shanghai, China). Briefly, after treatment with FLK or LCB, the cells (3.0 × 10^5^) were incubated with 2 μM Fluo-4 AM at 37 °C for 30 min. Then, the cells were washed three times with PBS and green fluorescence was observed under a fluorescence microscope. Afterward, the cells were collected and prepared as a single-cell suspension to detect the content of Ca^2+^ via flow cytometry (BD Biosciences, San Jose, CA, USA). Exactly 10,000 cellular events were captured and analyzed per sample.

### 2.15. Western Blot Assay

The treated cells were harvested and lysed in cell lysis buffer (Beyotime, Shanghai, China) for 1 h. Then, the concentration of protein was measured by the bicinchoninic acid (BCA) assay using a BCA protein concentration detection kit (Solarbio, Beijing, China). Next, equal amounts of cell lysates (50 µg protein) were separated via SDS-PAGE and transferred to a PVDF membrane (Merck, Darmstadt, Germany). All PVDF membranes were blocked with 5% non-fat milk (Yili, Hohhot, China) for 2 h at room temperature and then incubated with primary antibodies (Caspase-3, Cat# 9662S, 1:1000, Cell Signaling Technology, Danvers, MA, USA; GSDME, Cat# 13075-1-AP, 1:1000, Proteintech, Rosemont, IL, USA; P-P38, Cat# 4511S, 1:1000, Cell Signaling Technology, Danvers, MA, USA; P38, Cat# 8690S, 1:1000, Cell Signaling Technology, Danvers, MA, USA; P-JNK, Cat# 4668S, 1:1000, Cell Signaling Technology, Danvers, MA, USA; JNK, Cat# 9252S, 1:1000, Cell Signaling Technology, Danvers, MA, USA; p53, Cat# 345567, 1:1000, ZenBio, Chengdu, China; P-PERK, Cat# 340846, 1:1000, ZenBio, Chengdu, China; PERK, Cat# CY2759, 1:1000, Abways, Shanghai, China; GRP78, Cat# CY5167, 1:1000, Abways, Shanghai, China; P-EIF2α, Cat# CY5036, 1:1000, Abways, Shanghai, China; EIF2α, Cat# 340347, 1:1000, ZenBio, Chengdu, China; CHOP, Cat# CY6694, 1:1000, Abways, Shanghai, China; GAPDH, Cat# A19056, 1:1000, ABclonal, Wuhan, China) at 4 °C. After 12 h incubation, the membranes were washed three times with TBS and incubated with anti-rabbit IgG (H+L) (DyLight^®^ 800 4X PEG Conjugate) secondary antibody (Cat# 5151, 1:30,000, Cell Signaling Technology, Danvers, MA, USA) for 2 h at room temperature in the dark. Finally, blot images were captured via the Odyssey platform (LI-COR Biosciences, Lincoln, NE, USA).

### 2.16. Animal Experiments

Female BALB/c mice (6 weeks old, 16~18 g) were obtained from Beijing Sibeifu Biotechnology Co., LTD (Beijing, China). After adaptive feeding for a week, mice without observable abnormalities were used in the subsequent experiments. 4T1 cells in the logarithmic growth phase were resuspended in cold saline solution at a concentration of 1 × 10^6^ cells/mL. Then, the 50 μL of the cell suspension was injected into the left inguinal mammary fat pad. After tumor establishment, mice were randomly divided into six groups, including NC, low dose (468 mg/kg), medium dose (936 mg/kg), high dose (1872 mg/kg), high dose+CTX (1872 mg/kg + 30 mg/kg) and CTX (30 mg/kg). According to the group assignment, all mice were continuously treated for 26 days. Body weight and tumor size were recorded. Tumor volume (mm^3^) was calculated as follows: 1/2 × (the shortest diameter)^2^ × (the longest diameter). Finally, mice were sacrificed, and the tumor tissues were used for further analysis. The animal experiments were permitted by the Animal Care and Use Committee of Guizhou Medical University (Approval Number: 2101008).

### 2.17. Hematoxylin-Eosin (H&E) Staining

The H&E staining was conducted to analyze the histopathological characteristics of tumors. Briefly, tumor tissues were fixed by 4% paraformaldehyde (Beyotime, Shanghai, China) and then embedded in paraffin. Next, the paraffin was sliced into 4-μm-thick sections, which were sequentially stained by hematoxylin and eosin (Beyotime, Shanghai, China). Afterward, histopathological changes were observed under a light microscope (Leica Microsystems, Wetzlar, Germany).

### 2.18. Immunohistochemistry (IHC) Assay

The IHC assay was employed to detect Ki-67 expression in tumor tissues. After paraffin embedding and sectioning, tissue sections were subjected to heat-induced antigen retrieval with 1:10 diluted 10× Universal HIER antigen retrieval reagent (Abcam, Cambridge, UK) and then rinsed with deionized water, followed by blocking with 1% bovine serum albumin. Next, the tissue sections were stained with anti-Ki-67 antibody (Cat# ab15580, 1:200, Abcam, Cambridge, UK) and then with a secondary antibody (Cat# ab6721, 1:500, Abcam, Cambridge, UK). Finally, following staining with DAB (Abcam, Cambridge, UK) and hematoxylin solution (Abcam, Cambridge, UK), images were captured under a light microscope (Leica Microsystems, Wetzlar, Germany).

### 2.19. Statistical Analysis

Statistical analysis was performed with GraphPad Prism 8.0.2. All data are presented as mean ± SD from at least three independent experiments. The *t*-test, one-way ANOVA test and two-way ANOVA test were used to analyze the data. *p* < 0.05, <0.01 or <0.001 were considered statistically significant.

## 3. Results

### 3.1. FLK Inhibits the Proliferation of TNBC 2D Cell Lines and 3D Patient-Derived Organoids

**Figure 1:**
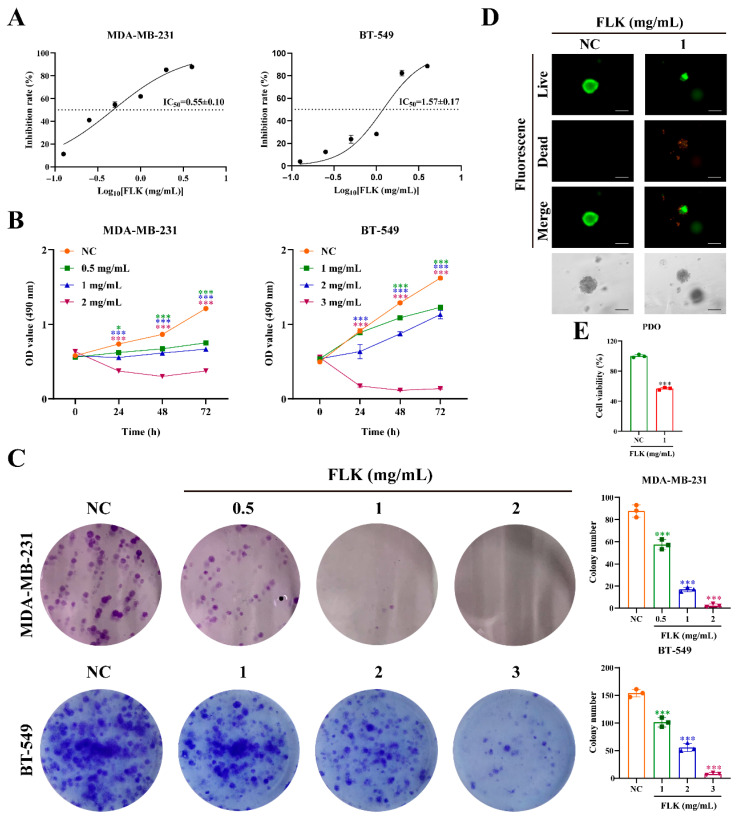
FLK inhibits the proliferation of TNBC 2D cell lines and 3D patient-derived organoids. (**A**) The IC_50_ values of FLK on MDA-MB-231 and BT-549 cells at 72 h. (**B**) An MTT assay was performed to detect cell viability of MDA-MB-231 and BT-549 cells treated with FLK at different time points. (**C**) The colony formation of MDA-MB-231 and BT-549 cells exposed to FLK. (**D**) The morphology of the organoids treated with FLK was observed (magnification: ×200; scale bar: 100 μm). (**E**) CCK-8 determined the cell viability of the organoids. Each datum represents the mean ± SD of three independent experiments. * *p* < 0.05, *** *p* < 0.001 vs. the NC group.

To evaluate whether FLK exerts anti-cancer effects on TNBC cells, we first assessed cell viability using the MTT assay. As shown in Figure 1A, FLK inhibited TNBC cell proliferation with IC_50_ values of 0.55 ± 0.10 mg/mL for MDA-MB-231 and 1.57 ± 0.17 mg/mL for BT-549 cells, respectively. Consistently, the cell growth curve further confirmed the inhibitory effect of FLK on TNBC cells (Figure 1B). Additionally, colony formation assays revealed that FLK markedly reduced both the number and size of colonies in a concentration-dependent manner (Figure 1C). Given that 3D organoids closely recapitulate the therapeutic responses of clinical tumors, FLK was also observed to maintain a significant inhibitory effect on tumor growth in patient-derived TNBC organoids (Figure 1D,E). Collectively, these results demonstrate that FLK suppresses TNBC cell proliferation in vitro and in patient-derived organoids, highlighting its potential as a novel therapeutic agent for TNBC.

### 3.2. FLK Induces Pyroptosis in TBNC Cells

**Figure 2:**
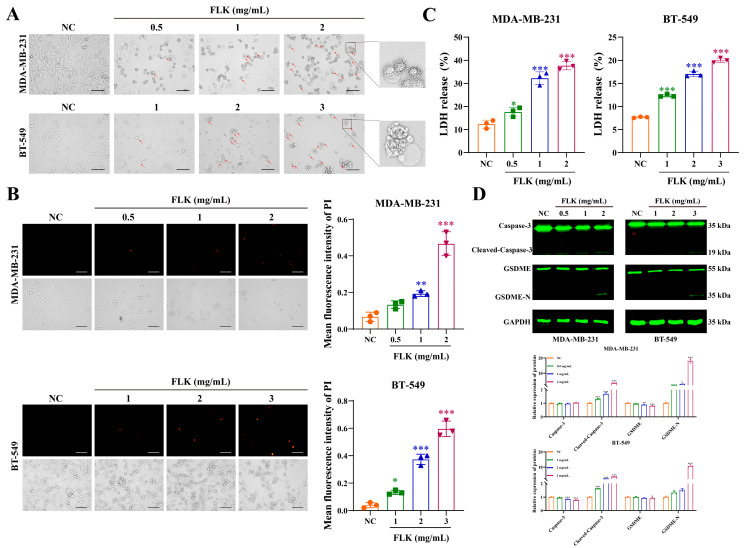
FLK induces pyroptosis in TBNC cells. (**A**) The morphologic change in MDA-MB-231 and BT-549 cells after treatment with FLK for 24 h under the light microscope: red arrowheads showed cell bubbling (magnification: ×200. Scale bar: 100 µm). (**B**) Representative PI fluorescence images of MDA-MB-231 and BT-549 cells treated with FLK for 24 h (magnification: ×200. Scale bar: 100 µm). (**C**) The release of LDH after treatment with FLK for 24 h in MDA-MB-231 and BT-549 cells. (**D**) After 24 h of FLK treatment, the expression of pyroptosis-related proteins was measured by Western blot in MDA-MB-231 and BT-549 cells. Each datum represents the mean ± SD of three independent experiments. * *p* < 0.05, ** *p* < 0.01, *** *p* < 0.001 vs. the NC group.

We next sought to clarify how FLK induces cell death. Morphological observation under light microscopy revealed that FLK-treated TNBC cells exhibited typical features of pyroptosis, including cell swelling and membrane blebbing (Figure 2A). To determine whether FLK disrupts membrane integrity, PI staining and LDH release assays were performed. The results showed that both the number of PI-positive cells (Figure 2B) and the level of LDH release (Figure 2C) increased in a concentration-dependent manner following FLK treatment. These results suggest that FLK may induce pyroptosis in TNBC cells.

GSDME, a key executor of pyroptosis, is cleaved and activated by Caspase-3 [23]. Therefore, we examined the expression of Cleaved-Caspase-3 and the N-terminal fragment of GSDME (GSDME-N) to investigate whether FLK induces pyroptosis via the Caspase-3/GSDME axis. As expected, Western blot analysis showed that the levels of Cleaved-Caspase-3 and GSDME-N were markedly increased in FLK-treated cells (Figure 2D). These findings indicate that FLK induces pyroptosis in TNBC cells through activation of the Caspase-3/GSDME pathway.

### 3.3. FLK Increases the Level of ROS and Decreases MMP in TNBC Cells

**Figure 3:**
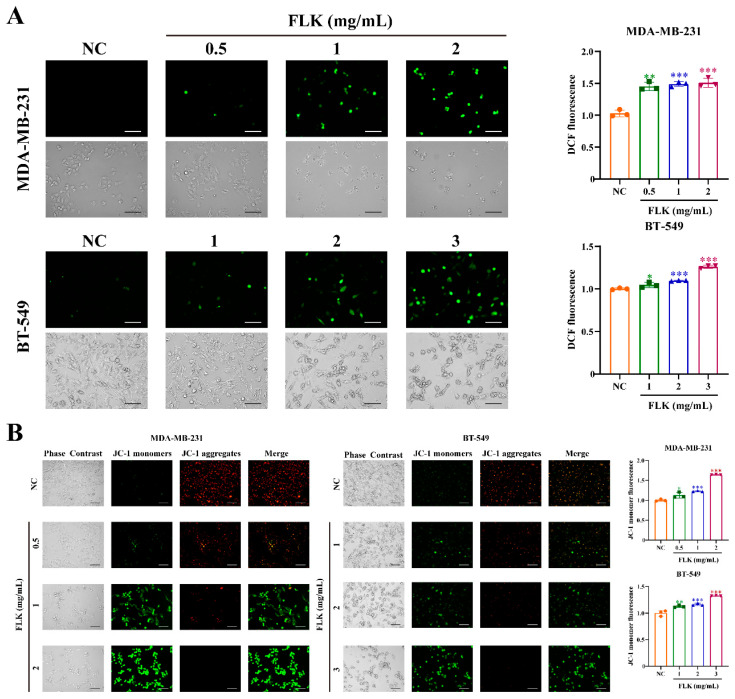
FLK increases the level of ROS and decreases MMP in TNBC cells. (**A**) The ROS level was measured by a reactive oxygen species assay kit, then fluorescence images and flow cytometry were used for qualitative and quantitative analysis of ROS, respectively, in MDA-MB-231 and BT-549 cells treated with FLK for 24 h (magnification: ×200; scale bar: 100 μm). (**B**) The change in MMP was shown by green fluorescence and red fluorescence, and the fluorescence intensity of JC-1 monomer/JC-1 aggregate was detected by flow cytometry in MDA-MB-231 and BT-549 cells treated with FLK for 24 h (magnification: ×200; scale bar: 100 μm). Each datum represents the mean ± SD of three independent experiments. * *p* < 0.05, ** *p* < 0.01, *** *p* < 0.001 vs. the NC group.

Mitochondrial dysfunction, which can activate the caspase cascade, has been closely linked to pyroptosis [24]. To investigate whether FLK triggers this pathway, we assessed ROS levels and MMP. Following FLK treatment, we observed a marked increase in intracellular ROS levels (Figure 3A). Concurrently, JC-1 staining revealed a decrease in MMP, as indicated by the increased proportion of JC-1 monomers (Figure 3B). These results demonstrate that FLK induces mitochondrial dysfunction by promoting ROS accumulation and disrupting MMP, which may in turn activate the caspase cascade to facilitate pyroptosis.

### 3.4. Transcriptomic Analysis Reveals Involvement of the Mitogen-Activated Protein Kinase (MAPK) Signaling Pathway in FLK-Induced Pyroptosis

**Figure 4:**
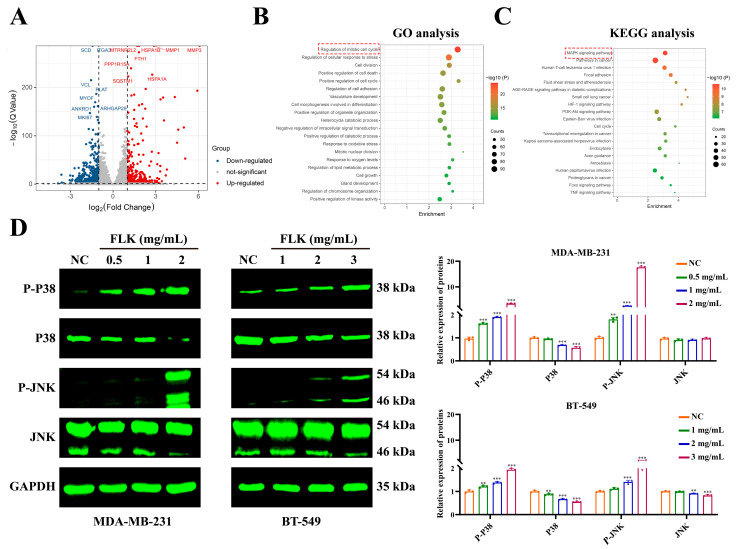
Transcriptomic analysis reveals involvement of the MAPK signaling pathway in FLK-induced pyroptosis. (**A**) The volcano plot of MDA-MB-231 treated with FLK for 24 h, including 553 upregulated genes (red dots) and 829 downregulated genes (blue dots). (**B**) The GO analysis of DEGs. (**C**) The top 20 enriched KEGG pathways of DEGs. (**D**) After treatment with FLK for 24 h, Western blot assay was utilized to analyze the expression of MAPK pathway-related proteins in MDA-MB-231 and BT-549 cells. Each datum represents the mean ± SD of three independent experiments. ** *p* < 0.01, *** *p* < 0.001 vs. the NC group.

To further elucidate the molecular mechanism underlying FLK-induced pyroptosis, we performed RNA-seq to profile global gene expression changes in FLK-treated TNBC cells. A total of 1382 DEGs were identified, comprising 553 upregulated and 829 downregulated genes (Figure 4A). GO enrichment analysis revealed that these DEGs were predominantly associated with biological processes such as cell cycle regulation, cell adhesion, and oxidative stress (Figure 4B). KEGG pathway analysis demonstrated significant enrichment of the MAPK signaling pathway, suggesting its potential involvement in FLK-mediated anti-tumor effects (Figure 4C).

Given that ROS accumulation has been reported to activate the P38/JNK axis, which consists of two vital stress-responsive MAPK proteins. The phosphorylation of P38 and JNK indicates pathway activation and mediates oxidative stress response as well as cell fate regulation [25,26]. We next investigated whether FLK engages this pathway in TNBC cells. Western blot analysis showed that FLK treatment increased the phosphorylation levels of P38 and JNK in a concentration-dependent manner (Figure 4D). Collectively, these findings suggest that FLK may trigger pyroptosis through ROS-mediated activation of the P38/JNK pathway, subsequently engaging the Caspase-3/GSDME cascade.

### 3.5. Identification of the Pharmacodynamic Basis and Network Pharmacology Analysis of FLK

**Figure 5:**
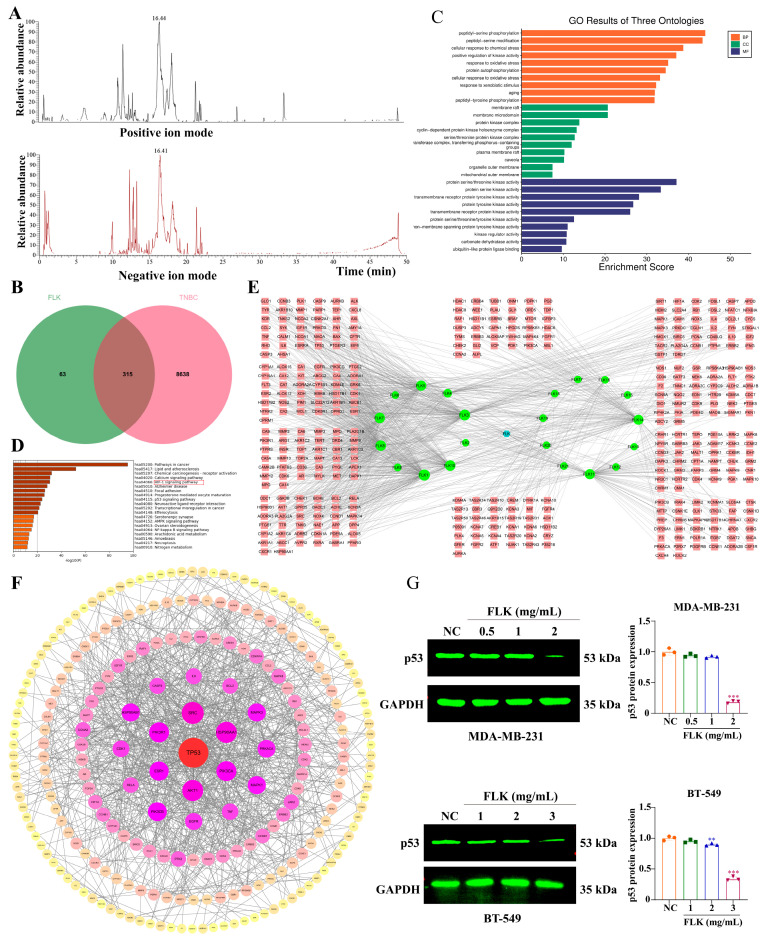
Identification of the pharmacodynamic basis and network pharmacology analysis of FLK. (**A**) UPLC-HR-MS/MS analysis of FLK in positive and negative ion modes. (**B**) A Venn diagram was used to analyze the overlapping genes. (**C**,**D**) The GO (**C**) and KEGG (**D**) analyses of overlapping genes. (**E**) FLK-compound-target network diagram (blue: FLK, green: ingredient, pink: target). (**F**) PPI network of 315 overlapping genes. (**G**) The expression of p53 in FLK-treated MDA-MB-231 and BT-549 cells. Each datum represents the mean ± SD of three independent experiments. ** *p* < 0.01, *** *p* < 0.001 vs. the NC group.

To investigate the active constituents underlying the anti-TNBC effect of FLK, we performed UPLC-HR-MS/MS analysis. A total of 96 chemical components were identified (Figure 5A) (Table S1). Based on their OB and DL values, 21 components with potential anti-cancer activity were selected for further analysis (Table S2). Subsequently, 1137 putative target genes for these 21 compounds were retrieved from the TCMSP and SwissADME databases (Table S3). Meanwhile, 8953 TNBC-related genes were obtained from DrugBank, GeneCards, OMIM, PharmGkb, and TTD databases. Venn diagram analysis revealed 315 intersecting genes between compound targets and TNBC-associated genes (Figure 5B).

GO and KEGG enrichment analyses were then performed on these 315 intersection genes. Consistent with our RNA-seq data, FLK was found to potentially regulate oxidative stress-related processes. Interestingly, the calcium signaling pathway emerged as a significantly enriched term (Figure 5C,D). We further constructed a compound-target network, which showed that several components—including FLK1 (Wogonin), FLK3 (Baicalein), FLK5 (Genkwanin), FLK7 (Pectolinarigenin), FLK8 (Oroxylin A), FLK10 (Morin), FLK11 (Praeruptorin A), and FLK14 (LCB)—targeted a greater number of genes compared to other constituents, suggesting their potential as major active contributors (Figure 5E). PPI network analysis further identified seven hub genes as potential therapeutic targets for TNBC, namely TP53, SRC, HSP90AA1, AKT1, PIK3R1, PIK3CA, and ESR1 (Figure 5F). Given the high mutation rate of TP53 (up to 80%) in TNBC and the well-established oncogenic role of mutant p53 in promoting tumor progression [27], we next investigated whether FLK modulates p53 expression. As shown in Figure 5G, FLK treatment markedly downregulated p53 protein levels in MDA-MB-231 and BT-549 cells.

### 3.6. FLK Induces Endoplasmic Reticulum Stress (ERS) via Calcium Dysregulation in TNBC Cells

**Figure 6:**
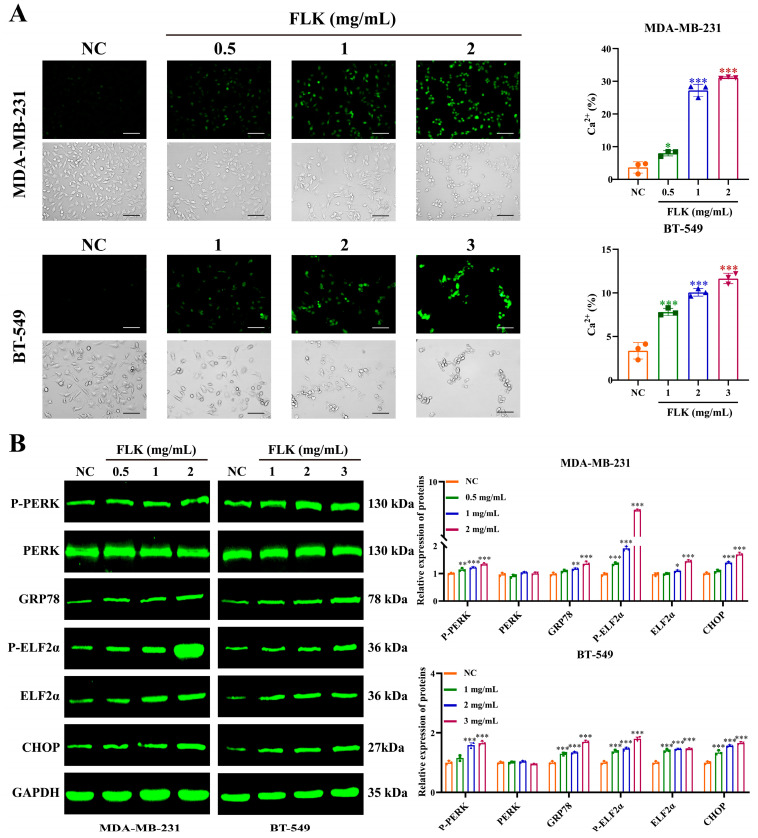
FLK induces ERS via calcium dysregulation in TNBC cells. (**A**) The level of Ca^2+^ was detected by Fluo-4 AM in MDA-MB-231 and BT-549 cells treated with FLK for 24 h (magnification: ×200; scale bar: 100 μm). (**B**) Western blot assay was used to analyze the expression of ERS-related proteins in MDA-MB-231 and BT-549 cells. Each datum represents the mean ± SD of three independent experiments. * *p* < 0.05, ** *p* < 0.01, *** *p* < 0.001 vs. the NC group.

Dysregulation of calcium homeostasis in the endoplasmic reticulum (ER) is a well-established trigger of ERS [28], and emerging evidence has linked ERS to the induction of pyroptosis [29]. Given that our network pharmacology analysis identified significant enrichment of the calcium signaling pathway, we hypothesized that FLK may disrupt calcium homeostasis, thereby inducing ERS in TNBC cells. To test this, we first measured intracellular calcium levels. As shown in Figure 6A, FLK treatment enhanced calcium release from the ER in a concentration-dependent manner. Consistently, Western blot analysis revealed that FLK activated the core ERS regulatory cascade of the PERK/GRP78/eIF2α/CHOP axis, where PERK and GRP78 act as vital ER stress sensors, while eIF2α and CHOP function as downstream effectors to transmit stress signals and trigger cell death, as evidenced by upregulated expression of these ERS-related proteins (Figure 6B). Collectively, these results suggest that FLK-induced pyroptosis may be mediated, at least in part, by calcium dysregulation and subsequent ERS.

### 3.7. FLK Suppresses Tumor Growth and Enhances Chemotherapeutic Efficacy in an Orthotopic TNBC Mouse Model

**Figure 7:**
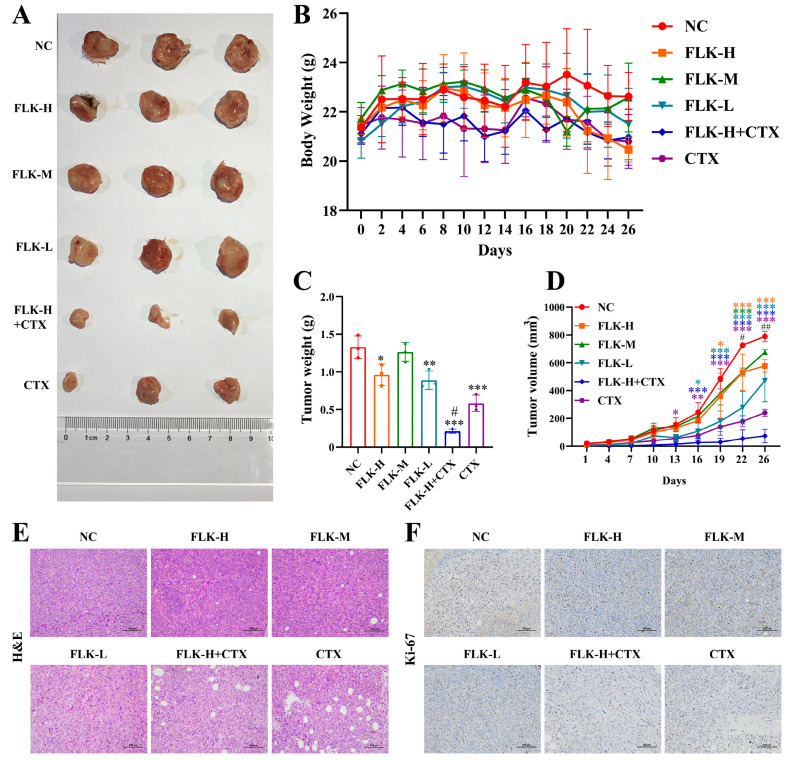
FLK suppresses tumor growth and enhances chemotherapeutic efficacy in an orthotopic TNBC mouse model. (**A**) Representative tumors of each group. (**B**) Body weight statistics of mice. (**C**) Tumor weight. (**D**) Tumor volume curve. (**E**) H&E staining of tumor sections (magnification: ×200; scale bar: 100 μm). (**F**) IHC staining of Ki-67 in tumor sections (magnification: ×200; scale bar: 100 μm). Each datum represents the mean ± SD. * *p* < 0.05, ** *p* < 0.01, and *** *p* < 0.001 vs. the NC group; ^#^ *p* < 0.05 and ^##^ *p* < 0.01 vs. the CTX group.

Building upon our in vitro findings demonstrating the anti-proliferative effects of FLK in TNBC cells and patient-derived organoids, we next evaluated its in vivo anti-tumor efficacy using a 4T1 orthotopic TNBC model. Given that CTX is widely used in TNBC treatment with well-documented therapeutic benefits [30,31], we also investigated whether combination therapy with FLK could enhance anti-tumor effects. As shown in Figure 7A–D, FLK monotherapy significantly reduced both tumor weight and volume compared to the untreated control group. Notably, the combination of FLK and CTX exhibited superior therapeutic efficacy relative to CTX treatment alone. H&E staining further revealed marked morphological changes in tumors from the combination group, including loosely arranged cells, disrupted tissue architecture, and evident vacuolar degeneration, indicative of enhanced cell death (Figure 7E). Ki-67 is a classic biomarker for evaluating cell proliferation ability in tumor tissues. Immunohistochemical analysis also showed a marked decrease in Ki-67-positive cells following FLK treatment (Figure 7F). Collectively, these results demonstrate that FLK not only suppresses TNBC tumor growth in vivo but also potentiates the therapeutic effect of CTX.

### 3.8. LCB Is Identified as a Major Active Constituent of FLK Targeting p53 in TNBC

**Figure 8:**
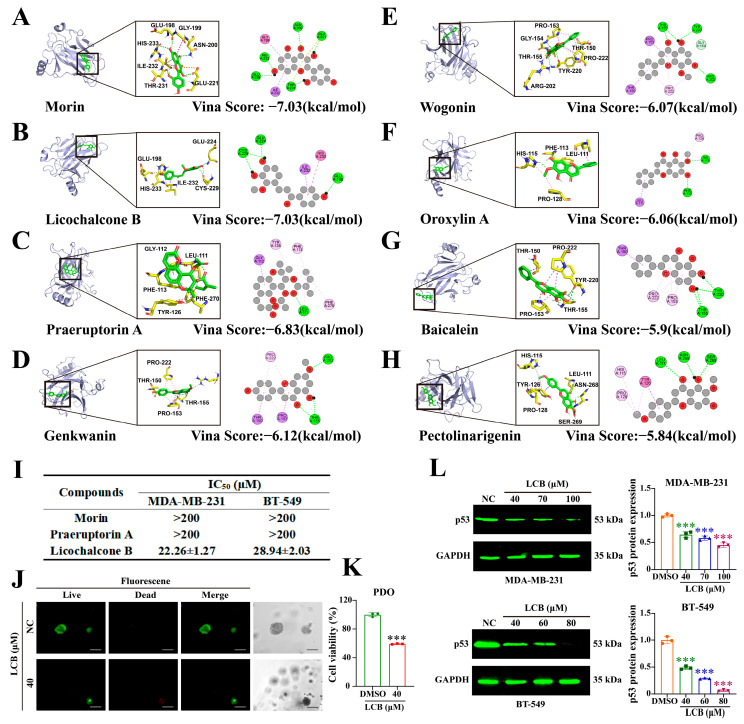
LCB is identified as a major active constituent of FLK targeting p53 in TNBC. (**A**–**H**) Molecular docking of the core components of FLK and the potential target p53. (**A**) Morin-p53, (**B**) LCB-p53, (**C**) Praeruptorin A-p53, (**D**) Genkwanin-p53, (**E**) Wogonin-p53, (**F**) Oroxylin A-p53, (**G**) Baicalein-p53, (**H**) Pectolinarigenin-p53. (**I**) IC_50_ of Morin, Praeruptorin A and LCB in TNBC cells. (**J**) The morphology of the organoids treated with LCB was observed (magnification: ×200; scale bar: 100 μm). (**K**) CCK-8 determined the cell viability of the organoids. (**L**) After treatment with LCB, the Western blot assay was used to analyze the expression of p53 in MDA-MB-231 and BT-549 cells. Each datum represents the mean ± SD of three independent experiments. *** *p* < 0.001 vs. the DMSO group.

To further investigate the interaction between the core components of FLK and the potential target p53, molecular docking analysis was performed. Among the eight candidate compounds—Wogonin, Baicalein, Genkwanin, Pectolinarigenin, Oroxylin A, Morin, Praeruptorin A, and LCB—Morin, LCB, and Praeruptorin A exhibited relatively high binding affinities to p53, with docking scores below −6.5 kcal/mol (Figure 8A–H). Subsequent cell viability assays revealed that LCB exerted the most potent inhibitory effect on both MDA-MB-231 and BT-549 cells, with IC_50_ values of 22.26 ± 1.27 and 28.94 ± 2.03 μM, respectively (Figure 8I). Moreover, LCB also significantly suppressed the growth of patient-derived TNBC organoids (Figure 8J,K). Importantly, consistent with the effects of FLK, LCB treatment markedly downregulated p53 protein expression in TNBC cells (Figure 8L). These findings suggest that LCB may serve as a key bioactive constituent of FLK, exerting anti-TNBC effects at least in part through modulation of p53.

### 3.9. LCB Recapitulates FLK-Induced Pyroptosis via Caspase-3/GSDME Activation and ERS in TNBC Cells

**Figure 9:**
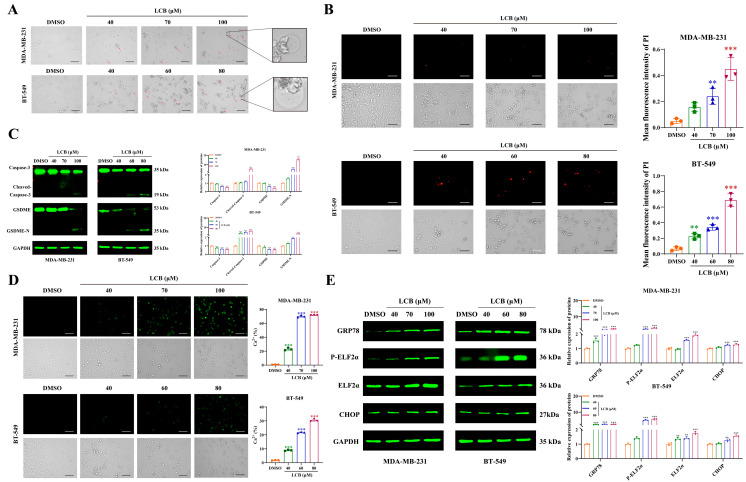
LCB recapitulates FLK-induced pyroptosis via Caspase-3/GSDME activation and ERS in TNBC cells. (**A**) The morphologic change in MDA-MB-231 and BT-549 cells after treatment with LCB under the light microscope: red arrowheads showed cell bubbling (magnification: ×200. Scale bar: 100 µm). (**B**) Representative PI fluorescence images of MDA-MB-231 and BT-549 cells treated with LCB (magnification: ×200. Scale bar: 100 µm). (**C**) The expression of pyroptosis-related proteins in LCB-treated MDA-MB-231 and BT-549 cells. (**D**) The level of Ca^2+^ was detected by Fluo-4 AM in MDA-MB-231 and BT-549 cells treated with LCB (magnification: ×200; Scale bar: 100 μm). (**E**) The expression of ERS-related proteins in LCB-treated MDA-MB-231 and BT-549 cells. Each datum represents the mean ± SD of three independent experiments. * *p* < 0.05, ** *p* < 0.01, *** *p* < 0.001 vs. the DMSO group.

To further validate whether LCB, identified as a key active constituent of FLK, reproduces the pyroptotic effects observed with FLK treatment, we examined its functional and molecular effects in TNBC cells. Morphological observation under light microscopy revealed that LCB treatment induced typical features of pyroptosis, including cell swelling and membrane blebbing, in both MDA-MB-231 and BT-549 cells (Figure 9A). Consistently, PI staining showed a concentration-dependent increase in PI-positive cells following LCB treatment, confirming LCB-induced membrane disruption (Figure 9B). Western blot analysis revealed that LCB activated the Caspase-3/GSDME cascade, as evidenced by elevated levels of Cleaved-Caspase-3 and GSDME-N, mirroring the effects of FLK (Figure 9C). Notably, LCB treatment also promoted calcium release and activated ERS, as indicated by upregulated expression of ERS-related proteins, consistent with the mechanistic profile of FLK (Figure 9D,E). Collectively, these results demonstrate that LCB recapitulates the pyroptotic phenotype induced by FLK in TNBC cells through activation of the Caspase-3/GSDME axis, likely mediated by calcium dysregulation and subsequent ERS.

## 4. Discussion

Pyroptosis has gained increasing attention as a therapeutic mechanism in cancer, particularly in tumors that exhibit resistance to apoptosis [32]. Previous studies have demonstrated that activation of the Caspase-3/GSDME axis can switch apoptosis to pyroptosis, thereby enhancing anti-tumor efficacy [23,33]. In the present study, our findings support this concept by demonstrating that FLK activates the Caspase-3/GSDME pathway in TNBC cells. This is consistent with previous reports showing that chemotherapeutic agents and natural compounds can induce pyroptosis through caspase-dependent mechanisms [34,35,36]. However, unlike most prior studies focusing on single agents, our results extend this paradigm to a clinically approved multi-component formulation, suggesting that coordinated regulation of multiple upstream signals may represent an alternative strategy to efficiently trigger pyroptosis.

One of the central observations of this study is that FLK-induced pyroptosis is associated with coordinated activation of ROS signaling and the MAPK pathway. ROS accumulation has been widely recognized as a critical upstream event in stress-induced cell death, including pyroptosis [37]. In TNBC, basal ROS levels are often elevated due to metabolic reprogramming and mitochondrial dysfunction, which can promote tumor progression but also render cancer cells more vulnerable to further oxidative stress [38]. In line with this concept, our data show that FLK treatment markedly increases intracellular ROS levels and disrupts mitochondrial membrane potential, indicating pronounced mitochondrial dysfunction. It should be noted that the MTT assay used in this study primarily reflects mitochondrial metabolic activity through succinate dehydrogenase (SDH), rather than directly measuring cell number. Therefore, the reduction in MTT signal following FLK treatment may, at least in part, reflect impaired mitochondrial function. Consistent with this interpretation, JC-1 staining revealed a significant loss of mitochondrial membrane potential, supporting the notion that FLK-induced mitochondrial dysfunction contributes to the decreased metabolic activity observed in the MTT assay. Given that mitochondrial damage is a key upstream event in caspase activation, these findings provide a mechanistic link between oxidative stress and the activation of downstream cell death pathways.

Previous studies have shown that oxidative stress-induced ROS generation can promote phosphorylation of P38 and JNK, leading to activation of downstream inflammatory pathways and pyroptosis [39,40]. Given that the P38/JNK signaling modules function as context-dependent regulators of survival and death in breast cancer, their activation under excessive stress conditions is generally associated with pro-death signaling [25,41]. Consistent with these observations, our transcriptomic and biochemical analyses demonstrate activation of the P38/JNK pathway in response to FLK treatment, supporting the notion that FLK drives TNBC cells beyond their oxidative stress threshold to initiate pyroptotic cell death. Taken together, these results suggest that FLK promotes pyroptosis in TNBC cells by inducing mitochondrial dysfunction and activating ROS-dependent MAPK signaling, ultimately engaging the Caspase-3/GSDME pathway.

In this regard, the involvement of calcium dysregulation and ERS represents a key mechanistic extension of current knowledge. While ERS has been increasingly linked to pyroptosis, its upstream triggers and integration with other stress pathways remain incompletely understood [42]. Calcium signaling is a central regulator of cellular homeostasis, and in cancer cells—including TNBC—its dysregulation contributes to proliferation, migration, and therapy resistance [43,44,45]. Notably, excessive intracellular calcium release can disrupt ER function, leading to accumulation of misfolded proteins and activation of the unfolded protein response [46,47]. Our network pharmacology analysis identified the calcium signaling pathway as significantly enriched, and subsequent validation demonstrated that FLK induces calcium release and activates the PERK/GRP78/EIF2α/CHOP axis. This sequence of events suggests that FLK-induced calcium imbalance serves as an initiating signal that triggers ERS, which can further amplify cell death signaling through crosstalk with mitochondrial dysfunction and caspase activation. These observations are consistent with previous reports showing that ERS functions as a signaling hub linking metabolic disturbance to inflammatory forms of cell death, including pyroptosis [48,49]. Taken together, our results support a model in which calcium dysregulation and ERS cooperate with ROS signaling to drive activation of the Caspase-3/GSDME pathway.

Another important finding is the identification of LCB as a key bioactive component contributing to the effects of FLK. LCB, a flavonoid extracted from the roots of *Glycyrrhiza inflata*, has been reported to exert anti-tumor activities through modulation of oxidative stress and apoptotic signaling in various cancers, such as colorectal cancer [50], oral squamous cell carcinoma [51], non-small-cell lung cancer [52] and bladder cancer [53]. However, its role in regulating pyroptosis remains less well characterized. Our data show that LCB recapitulates the major phenotypic and molecular effects of FLK, including activation of the Caspase-3/GSDME axis and induction of ERS. In addition, both FLK and LCB were found to downregulate p53 protein levels. Given that mutant p53 is frequently stabilized in TNBC and contributes to tumor progression and therapeutic resistance [54]. Therefore, a reduction in mutant p53 may lower the threshold for stress-induced cell death. Given that p53 has been implicated in regulating cellular responses to oxidative and ERS, its downregulation may further sensitize TNBC cells to FLK-induced pyroptotic signaling [55,56]. Although the precise link between p53 modulation and pyroptosis requires further investigation, these findings raise the possibility that targeting mutant p53 may facilitate the induction of inflammatory cell death.

An important consideration in the therapeutic application of pyroptosis is its dual role in cancer [57]. While pyroptosis can promote anti-tumor immunity through the release of inflammatory mediators, excessive or chronic inflammation may conversely support tumor progression and metastasis [58,59]. In breast cancer, particularly in apoptosis-resistant subtypes such as TNBC, pyroptosis is increasingly considered a therapeutically relevant form of cell death; however, its benefit is highly context-dependent [60]. Transient activation may enhance anti-tumor immunity, whereas sustained inflammation could potentially promote tumor aggressiveness. Therefore, the net outcome of pyroptosis induction is likely determined by the balance between immune activation and pro-tumorigenic inflammation. In our study, the overall effects of FLK were consistently tumor-suppressive across in vitro and in vivo models, and combination treatment further enhanced the efficacy of chemotherapy. These findings suggest that, under the conditions tested, FLK-induced pyroptosis favors anti-tumor outcomes. Nevertheless, the impact of FLK on the tumor immune microenvironment and long-term inflammatory responses remains to be defined and represents an important direction for future research.

Finally, the multi-component nature of FLK may provide a mechanistic basis for its ability to orchestrate complex cell death signaling networks. Unlike single-molecule agents, TCM formulations contain multiple bioactive constituents that can simultaneously modulate interconnected pathways, including oxidative stress, calcium signaling, and ERS [61]. Such multi-target regulation may be particularly advantageous in TNBC, where redundant survival pathways often limit the efficacy of single-target therapies [62]. This systems-level regulation may be particularly effective for inducing pyroptosis, which requires coordinated activation of multiple upstream signals. Our findings therefore not only provide insight into the anti-TNBC activity of FLK but also highlight the potential advantages of multi-component therapeutics in targeting complex cell death mechanisms.

Several limitations should be acknowledged. First, although our results support a tumor-suppressive role of FLK-induced pyroptosis, the contribution of immune responses and the broader impact on the tumor microenvironment were not directly evaluated. Second, given the dual role of pyroptosis-associated inflammation, the potential consequences of sustained inflammatory signaling on tumor progression remain to be further clarified. Third, although FLK demonstrated clear anti-tumor efficacy in vivo, the direct effects of the isolated active compound LCB have not yet been validated in animal models. Future in vivo studies focusing on LCB will be important to determine whether it can recapitulate the therapeutic efficacy of the full formulation and to further strengthen the translational relevance of our findings. Addressing these limitations will be essential for a more comprehensive understanding of the therapeutic potential of FLK.

## 5. Conclusions

**Figure 10:**
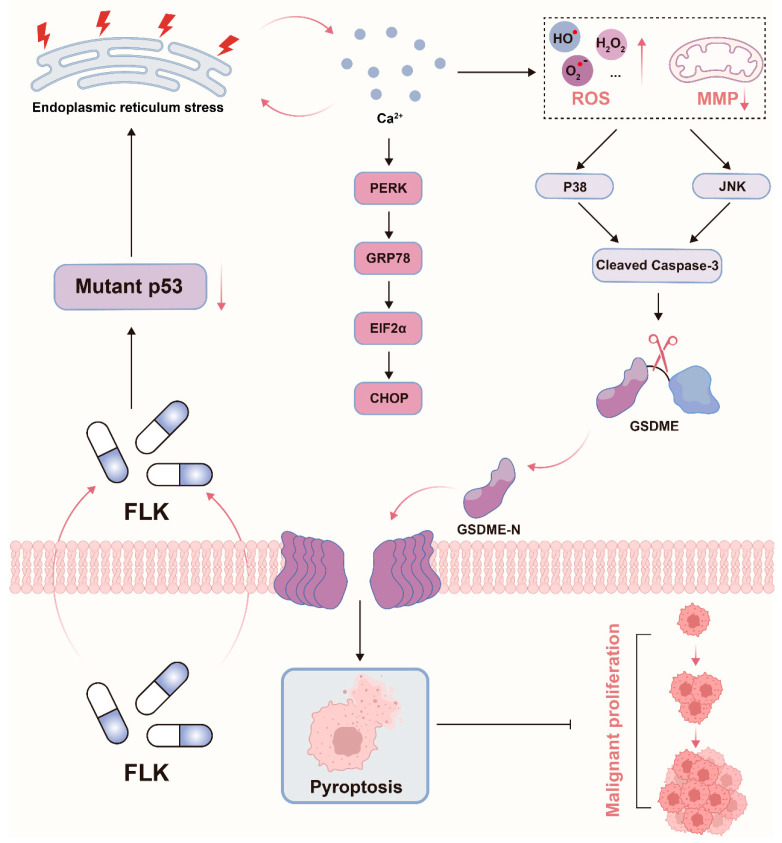
FLK induces pyroptosis in TNBC cells via mutant p53-calcium/ER stress-ROS/P38-JNK-mediated Caspase-3/GSDME cascade.

In conclusion, this study elucidates a novel mechanism by which FLK induces pyroptosis in TNBC cells through a “p53-calcium dysregulation-ER stress-mitochondrial damage-Caspase-3/GSDME” cascade (Figure 10). In addition, LCB was identified as a key bioactive component that recapitulates the major effects of FLK. These findings provide mechanistic insight into the anti-tumor activity of this multi-component formulation and suggest that targeting pyroptosis may represent a promising therapeutic strategy for TNBC.

## Data Availability

The original contributions presented in this study are included in the article/Supplementary Materials. Further inquiries can be directed to the corresponding author(s). RNA sequence data have been officially released under the accession number HRA018526 in the GSA-Human Data Access Committee (DAC).

## Supplementary Materials

The following supporting information can be downloaded at https://www.mdpi.com/article/10.3390/antiox15050649/s1, Table S1. Chemical components in FLK identified by UPLC-HR-MS/MS. Table S2. The oral bioavailability and drug-likeness of 21 components. Table S3. The 1137 target genes of 21 components.

## Abbreviations

Bicinchoninic acid, BCA; Cell Counting Kit-8, CCK-8; Cyclophosphamide, CTX; Differential expression genes, DEGs; Dimethyl sulfoxide, DMSO; 3-(4,5-dimethylthiazol-2-yl)-2,5-diphenyltetrazolium bromide, MTT; Drug-likeness, DL; Electrospray ionization, ESI; Endoplasmic reticulum stress, ERS; Gene Ontology, GO; Hematoxylin-Eosin, HE; Hormone receptor-positive, HR; Human epidermal growth factor receptor 2-positive, HER-2; Immunohistochemistry, IHC; Kyoto Encyclopedia of Genes and Genomes, KEGG; Lactate Dehydrogenase, LDH; Licochalcone B, LCB; Mitochondrial Membrane Potential, MMP; Mitogen-Activated Protein Kinase, MAPK; Oral bioavailability, OB; Orbitrap Traditional Chinese Medicine Library, OTCML; Phosphate-buffered solution, PBS; Propidium iodide, PI; Protein–protein interaction, PPI; Reactive Oxygen Species, ROS; RNA sequencing, RNA-seq; Succinate dehydrogenase, SDH; Traditional Chinese Medicine, TCM; Triple-negative breast cancer, TNBC; Ultra-Performance Liquid Chromatography–High-Resolution Tandem Mass Spectrometry, UPLC-HR-MS/MS.
