# Supplementary material for: Feilike and Its Constituent Licochalcone B Trigger Caspase-3/GSDME-Mediated Pyroptosis in Triple-Negative Breast Cancer via Modulation of the Mutant p53–Calcium/ER Stress–ROS–MAPK Axis
Source: Antioxidants (Basel). 2026 May 21;15(5):649. doi: 10.3390/antiox15050649 (PMC13203176; doi:10.3390/antiox15050649)
Supplement: Supplementary file 1 [file antioxidants-15-00649-s001.zip › Table S1.pdf]

Table S1. Chemical components in FLK identified by UPLC-HR-MS/MS

Serial number	t_R/min	Name	Formula	Molecular Weight
1	0.707	Mannitol	C6 H14 O6	182.07817
2	0.88	Maleic acid	C4 H4 O4	116.00973
3	1.6	L-Phenylalanine	C9 H11 N O2	165.07871
4	1.826	5-Hydroxymethylfurfural	C6 H6 O3	126.03176
5	2.598	Feretoside	C17 H24 O11	440.10817
6	2.734	Gardenoside	C17 H24 O11	404.13148
7	3.809	Forsythoside E	C20 H30 O12	462.17359
8	3.837	Protocatechualdehyde	C7 H6 O3	138.03056
9	4.114	Orcinol gentiobioside	C19 H28 O12	448.15782
10	4.206	Salicylic acid	C7 H6 O3	138.03056
11	4.307	Hastatoside	C17 H24 O11	450.13722
12	5.46	Secoxyloganin	C17 H24 O11	404.13134
13	5.909	Loganic acid	C16 H24 O10	376.13682
14	6.221	Lawsone	C10 H6 O3	174.03151
15	6.225	p-Hydroxybenzaldehyde	C7 H6 O2	122.0356
16	6.459	Esculetin	C9 H6 O4	178.02595
17	6.702	Vanillic acid	C8 H8 O4	168.04199
18	6.719	Asperulosidic acid	C18 H24 O12	432.1266
19	7.289	Caffeic acid	C9 H8 O4	180.04141
20	7.849	1-Caffeoylquinic acid	C16 H18 O9	354.09493
21	7.856	Quinic acid	C7 H12 O6	192.06265
22	8.427	Neomangiferin	C25 H28 O16	584.13723
23	8.492	Asperuloside	C18 H22 O11	460.1215
24	9.053	Cryptochlorogenic acid	C16 H18 O9	354.09497
25	9.111	Scopoletin	C10 H8 O4	192.04199
26	9.339	2-Hydroxy-4-methoxybenzaldehyde	C8 H8 O3	152.04714
27	9.348	Taxifolin	C15 H12 O7	304.05823
28	9.694	Isopsoralen	C11 H6 O3	186.03159
29	9.901	Sweroside	C16 H22 O9	358.12586
30	9.996	7,8-Dihydroxycoumarin	C9 H6 O4	178.02576
31	10.275	Sibiricose A5	C22 H30 O14	518.16334
32	10.342	Coumarin	C9 H6 O2	146.03659
33	10.496	Shikimic acid	C7 H10 O5	174.05189
34	10.5	Vicenin II	C27 H30 O15	594.15809
35	10.506	Cynaroside	C21 H20 O11	448.10005
36	10.572	Albiflorin	C23 H28 O11	526.16829
37	10.675	2''-O-β-L-Galactopyranosylorientin	C27 H30 O16	610.15287
38	10.824	Morin	C15 H10 O7	302.04216
39	10.985	7-Hydroxycoumarin	C9 H6 O3	162.03109
40	11.103	Homoorientin	C21 H20 O11	448.09996
41	11.307	Schaftoside	C26 H28 O14	564.14725

42	11.324	Isoschaftoside	C26 H28 O14	564.1472
43	11.618	4'-O-Glucosylvitexin	C27 H30 O15	594.15756
44	11.807	Isomangiferin	C19 H18 O11	422.08467
45	12.027	Secoisolariciresinol diglucoside	C32 H46 O16	686.27809
46	12.221	Rutin	C27 H30 O16	610.15318
47	12.365	Hyperoside	C21 H20 O12	464.09528
48	12.493	Cimifugin	C16 H18 O6	306.11012
49	12.698	Verbascoside	C29 H36 O15	624.20507
50	12.756	Astringin	C20 H22 O9	406.12569
51	12.757	Kaempferol-3-O-rutinoside	C27 H30 O15	594.15776
52	12.875	Naringin dihydrochalcone	C27 H34 O14	582.19478
53	13.086	Puerarin	C21 H20 O9	416.11055
54	13.12	Poncirin	C28 H34 O14	594.19404
55	13.146	Tuberostemonine	C22 H33 N O4	375.2404
56	13.147	Aurantio-obtusin β-D-glucoside	C23 H24 O12	492.12633
57	13.221	Isoacteoside	C29 H36 O15	624.20507
58	13.263	Skimmin	C15 H16 O8	324.08418
59	13.268	Astragalin	C21 H20 O11	448.1003
60	13.381	Kaempferol	C15 H10 O6	286.04729
61	13.386	5-O-Methylvisammioside	C22 H28 O10	452.16792
62	13.45	Azelaic acid	C9 H16 O4	188.104
63	13.555	Isosakuranetin	C16 H14 O5	286.08336
64	13.694	Luteolin	C15 H10 O6	286.04742
65	13.727	Isochlorogenic acid B	C25 H24 O12	516.12642
66	13.809	Scutellarin	C21 H18 O12	462.07968
67	13.885	Decursinol	C14 H14 O4	246.08899
68	14.122	Isochlorogenic acid C	C25 H24 O12	516.1264
69	14.363	Marmesin	C14 H14 O4	246.08899
70	14.937	Homoplantaginin	C22 H22 O11	462.11584
71	15.057	Scutellarin methyl ester	C22 H20 O12	476.09508
72	15.548	Glycitin	C22 H22 O10	446.12079
73	15.641	Genkwanin	C16 H12 O5	284.06809
74	15.794	Brazilin	C16 H14 O5	286.08348
75	15.903	Baicalein	C15 H10 O5	270.05272
76	16.505	Oroxin A	C21 H20 O10	432.10472
77	17.475	Oxyresveratrol	C14 H12 O4	244.07326
78	17.54	Oroxylin A-7-O-β-D-glucuronide	C22 H20 O11	460.10034
79	18.137	Baicalin	C21 H18 O11	446.08455
80	18.158	Cardamoni	C16 H14 O4	270.08855
81	18.428	Tectorigenin	C16 H12 O6	300.06313
82	18.456	Iristectorigenin B	C17 H14 O7	330.07373
83	18.667	Wogonoside	C22 H20 O11	460.10026
84	19.122	Hispidulin	C16 H12 O6	300.06314
85	20.207	Jaceosidin	C17 H14 O7	330.07381

86	20.84	Irigenin	C18 H16 O8	360.08401
87	21.419	Wogonin	C16 H12 O5	284.06814
88	21.612	Chrysin	C15 H10 O4	254.05749
89	21.737	Pectolinarigenin	C17 H14 O6	314.07854
90	21.967	Chrysosplenetin B	C19 H18 O8	374.09974
91	22.143	Oroxylin A	C16 H12 O5	284.06812
92	22.871	Lysionotin	C18 H16 O7	344.08905
93	24.563	Rubiadin	C15 H10 O4	254.0576
94	24.809	5-Hydroxy-6,7-dimethoxylflavone	C17 H14 O5	298.08339
95	26.743	Licochalcone B	C16 H14 O5	286.08342
96	27.059	Praeruptorin A	C21 H22 O7	403.16216
