# Supplementary material for: Feilike and Its Constituent Licochalcone B Trigger Caspase-3/GSDME-Mediated Pyroptosis in Triple-Negative Breast Cancer via Modulation of the Mutant p53–Calcium/ER Stress–ROS–MAPK Axis
Source: Antioxidants (Basel). 2026 May 21;15(5):649. doi: 10.3390/antiox15050649 (PMC13203176; doi:10.3390/antiox15050649)
Supplement: Supplementary file 1 [file antioxidants-15-00649-s001.zip › Table S2.pdf]

Table S2. The oral bioavailablity and drug-likeness of 21 components

Serial number	Component	MOL ID	OB (%)	DL
1	Wogonin	MOL000173	30.68	0.23
2	Hispidulin	MOL001735	30.97	0.27
3	Baicalein	MOL002714	33.52	0.21
4	Luteolin	MOL000006	36.16	0.25
5	Genkwanin	MOL005573	37.13	0.24
6	Baicalin	MOL002776	40.12	0.75
7	Pectolinarigenin	MOL005842	41.17	0.30
8	Oroxylin A	MOL002928	41.37	0.23
9	Kaempferol	MOL000422	41.88	0.24
10	Morin	MOL000737	46.23	0.27
11	Praeruptorin A	MOL013079	46.46	0.53
12	Marmesin	MOL001944	50.28	0.18
13	Taxifolin	MOL004576	57.84	0.27
14	Licochalcone B	MOL004841	76.76	0.19
15	Brazilin	73384	High	0.55
16	Cardamoni	641785	High	0.55
17	Iristectorigenin B	5488781	High	0.55
18	7,8-Dihydroxycoumarin	5280569	High	0.55
19	5-Hydroxy-6,7-dimethoxylflavone	471722	High	0.55
20	Isosakuranetin	160481	High	0.55
21	Skimmin	99693	High	0.55
