# Supplementary material for: Feilike and Its Constituent Licochalcone B Trigger Caspase-3/GSDME-Mediated Pyroptosis in Triple-Negative Breast Cancer via Modulation of the Mutant p53–Calcium/ER Stress–ROS–MAPK Axis
Source: Antioxidants (Basel). 2026 May 21;15(5):649. doi: 10.3390/antiox15050649 (PMC13203176; doi:10.3390/antiox15050649)
Supplement: Supplementary file 1 [file antioxidants-15-00649-s001.zip › Table S3.pdf]

Table S3. The 1137 target genes of 21 components

Component	MOL ID	Target gene
Wogonin	MOL000173	PTGS2
Wogonin	MOL000173	NOS2
Wogonin	MOL000173	FLT3
Wogonin	MOL000173	AKR1B1
Wogonin	MOL000173	OPRD1
Wogonin	MOL000173	KIT
Wogonin	MOL000173	ABCB1
Wogonin	MOL000173	IKBKB
Wogonin	MOL000173	NTRK2
Wogonin	MOL000173	KDM4E
Wogonin	MOL000173	XDH
Wogonin	MOL000173	ALOX15
Wogonin	MOL000173	CDK1
Wogonin	MOL000173	ALOX12
Wogonin	MOL000173	GRK6
Wogonin	MOL000173	CYP19A1
Wogonin	MOL000173	ESR2
Wogonin	MOL000173	CYP1A1
Wogonin	MOL000173	OPRM1
Wogonin	MOL000173	ABCG2
Wogonin	MOL000173	ESR1
Wogonin	MOL000173	EGFR
Wogonin	MOL000173	SLC22A12
Wogonin	MOL000173	CYP1B1
Wogonin	MOL000173	CA4
Wogonin	MOL000173	CA2
Wogonin	MOL000173	CA1
Wogonin	MOL000173	MCL1
Wogonin	MOL000173	PIK3CG
Wogonin	MOL000173	PIM1
Wogonin	MOL000173	ADORA1
Wogonin	MOL000173	ADORA2A
Wogonin	MOL000173	HSD17B2
Wogonin	MOL000173	HSD17B1
Wogonin	MOL000173	CA7
Wogonin	MOL000173	CA12
Wogonin	MOL000173	CDK5R1
Wogonin	MOL000173	CCNB3
Wogonin	MOL000173	CDK6
Wogonin	MOL000173	CA9
Wogonin	MOL000173	CBR1
Wogonin	MOL000173	TERT

Wogonin	MOL000173	AR
Wogonin	MOL000173	CA6
Wogonin	MOL000173	PTPRS
Wogonin	MOL000173	DAPK1
Wogonin	MOL000173	MPG
Wogonin	MOL000173	PFKFB3
Wogonin	MOL000173	MMP9
Wogonin	MOL000173	MMP2
Wogonin	MOL000173	LCK
Wogonin	MOL000173	MMP12
Wogonin	MOL000173	CD38
Wogonin	MOL000173	TOP1
Wogonin	MOL000173	ARG1
Wogonin	MOL000173	MAPT
Wogonin	MOL000173	TOP2A
Wogonin	MOL000173	INSR
Wogonin	MOL000173	DRD4
Wogonin	MOL000173	MYLK
Wogonin	MOL000173	MPO
Wogonin	MOL000173	PIK3R1
Wogonin	MOL000173	PYGL
Wogonin	MOL000173	MMP13
Wogonin	MOL000173	MMP3
Wogonin	MOL000173	CA3
Wogonin	MOL000173	CA14
Wogonin	MOL000173	MET
Wogonin	MOL000173	CA13
Wogonin	MOL000173	CAMK2B
Wogonin	MOL000173	PLA2G1B
Wogonin	MOL000173	CA5A
Wogonin	MOL000173	APEX1
Wogonin	MOL000173	AKR1C2
Wogonin	MOL000173	AKR1C1
Wogonin	MOL000173	AKR1C3
Wogonin	MOL000173	AKR1C4
Wogonin	MOL000173	AKR1A1
Wogonin	MOL000173	GPR35
Wogonin	MOL000173	ODC1
Wogonin	MOL000173	HSP90AA1
Wogonin	MOL000173	ALOX5
Wogonin	MOL000173	CXCR1
Wogonin	MOL000173	PLA2G2A
Wogonin	MOL000173	SRC
Wogonin	MOL000173	APP

Wogonin	MOL000173	CYP1A2
Wogonin	MOL000173	ADORA3
Wogonin	MOL000173	HSP90B1
Wogonin	MOL000173	ABCC1
Wogonin	MOL000173	TNKS
Wogonin	MOL000173	TTR
Wogonin	MOL000173	NOX4
Wogonin	MOL000173	AVPR2
Wogonin	MOL000173	NAE1
Wogonin	MOL000173	BCHE
Wogonin	MOL000173	ACHE
Wogonin	MOL000173	BACE1
Wogonin	MOL000173	PDE5A
Wogonin	MOL000173	GSK3B
Wogonin	MOL000173	NOS2
Wogonin	MOL000173	PTGS1
Wogonin	MOL000173	PTGS2
Wogonin	MOL000173	ESR1
Wogonin	MOL000173	AR
Wogonin	MOL000173	SCN5A
Wogonin	MOL000173	PPARG
Wogonin	MOL000173	RXRA
Wogonin	MOL000173	DPP4
Wogonin	MOL000173	MAPK14
Wogonin	MOL000173	GSK3B
Wogonin	MOL000173	PIK3CG
Wogonin	MOL000173	CHEK1
Wogonin	MOL000173	ADRB2
Wogonin	MOL000173	GABRA1
Wogonin	MOL000173	RELA
Wogonin	MOL000173	AKT1
Wogonin	MOL000173	CCND1
Wogonin	MOL000173	BCL2
Wogonin	MOL000173	CDKN1A
Wogonin	MOL000173	EIF6
Wogonin	MOL000173	BAX
Wogonin	MOL000173	CASP9
Wogonin	MOL000173	KDR
Wogonin	MOL000173	TNF
Wogonin	MOL000173	IL6
Wogonin	MOL000173	AHSA1
Wogonin	MOL000173	CASP3
Wogonin	MOL000173	TP53
Wogonin	MOL000173	TEP1

Wogonin	MOL000173	MMP1
Wogonin	MOL000173	CCL2
Wogonin	MOL000173	PRKCD
Wogonin	MOL000173	PTGER3
Wogonin	MOL000173	FN1
Wogonin	MOL000173	CXCL8
Wogonin	MOL000173	MCL1
Hispidulin	MOL001735	NOS2
Hispidulin	MOL001735	PTGS1
Hispidulin	MOL001735	PTGS2
Hispidulin	MOL001735	DPP4
Hispidulin	MOL001735	PIK3CG
Hispidulin	MOL001735	NCOA2
Hispidulin	MOL001735	NCOA1
Hispidulin	MOL001735	ACHE
Hispidulin	MOL001735	RHO
Baicalein	MOL002714	KDM4E
Baicalein	MOL002714	XDH
Baicalein	MOL002714	ALOX15
Baicalein	MOL002714	CDK1
Baicalein	MOL002714	ALOX12
Baicalein	MOL002714	GRK6
Baicalein	MOL002714	CYP19A1
Baicalein	MOL002714	CA7
Baicalein	MOL002714	CA12
Baicalein	MOL002714	CA4
Baicalein	MOL002714	ABCB1
Baicalein	MOL002714	CYP1B1
Baicalein	MOL002714	HSD17B1
Baicalein	MOL002714	AKR1B1
Baicalein	MOL002714	CDK5R1
Baicalein	MOL002714	CA2
Baicalein	MOL002714	CCNB3
Baicalein	MOL002714	CDK6
Baicalein	MOL002714	CA1
Baicalein	MOL002714	CA9
Baicalein	MOL002714	ABCG2
Baicalein	MOL002714	CBR1
Baicalein	MOL002714	ESR2
Baicalein	MOL002714	ESR1
Baicalein	MOL002714	ACHE
Baicalein	MOL002714	ADORA1
Baicalein	MOL002714	ADORA2A
Baicalein	MOL002714	PTGS2

Baicalein	MOL002714	PTPRS
Baicalein	MOL002714	AMY1A
Baicalein	MOL002714	FLT3
Baicalein	MOL002714	IKBKB
Baicalein	MOL002714	NTRK2
Baicalein	MOL002714	AR
Baicalein	MOL002714	NOX4
Baicalein	MOL002714	MAOA
Baicalein	MOL002714	SYK
Baicalein	MOL002714	GSK3B
Baicalein	MOL002714	ABCC1
Baicalein	MOL002714	TTR
Baicalein	MOL002714	CSNK2A1
Baicalein	MOL002714	CFTR
Baicalein	MOL002714	AKR1B10
Baicalein	MOL002714	TNKS2
Baicalein	MOL002714	TNKS
Baicalein	MOL002714	HSD17B2
Baicalein	MOL002714	BCHE
Baicalein	MOL002714	ADORA3
Baicalein	MOL002714	TERT
Baicalein	MOL002714	LCK
Baicalein	MOL002714	PFKFB3
Baicalein	MOL002714	PIM1
Baicalein	MOL002714	PARP1
Baicalein	MOL002714	ALOX5
Baicalein	MOL002714	APP
Baicalein	MOL002714	CALM1
Baicalein	MOL002714	ARG1
Baicalein	MOL002714	NOS2
Baicalein	MOL002714	GLO1
Baicalein	MOL002714	MMP9
Baicalein	MOL002714	MMP2
Baicalein	MOL002714	MMP12
Baicalein	MOL002714	CD38
Baicalein	MOL002714	TOP1
Baicalein	MOL002714	NAE1
Baicalein	MOL002714	EGFR
Baicalein	MOL002714	SRC
Baicalein	MOL002714	TYR
Baicalein	MOL002714	AHR
Baicalein	MOL002714	ESRRA
Baicalein	MOL002714	KIT
Baicalein	MOL002714	OPRD1

Baicalein	MOL002714	CYP1A1
Baicalein	MOL002714	AURKB
Baicalein	MOL002714	PIK3CG
Baicalein	MOL002714	MAPT
Baicalein	MOL002714	TOP2A
Baicalein	MOL002714	INSR
Baicalein	MOL002714	MYLK
Baicalein	MOL002714	APEX1
Baicalein	MOL002714	IGF1R
Baicalein	MOL002714	KDR
Baicalein	MOL002714	PLK1
Baicalein	MOL002714	MET
Baicalein	MOL002714	ALK
Baicalein	MOL002714	AXL
Baicalein	MOL002714	PTPN1
Baicalein	MOL002714	ST6GAL1
Baicalein	MOL002714	SLC22A12
Baicalein	MOL002714	GPR35
Baicalein	MOL002714	FYN
Baicalein	MOL002714	TACR2
Baicalein	MOL002714	PRKDC
Baicalein	MOL002714	MAPK3
Baicalein	MOL002714	BACE1
Baicalein	MOL002714	SIRT1
Baicalein	MOL002714	PLA2G2A
Baicalein	MOL002714	PLA2G4A
Baicalein	MOL002714	CA6
Baicalein	MOL002714	CDK2
Baicalein	MOL002714	PTGS1
Baicalein	MOL002714	PTGS2
Baicalein	MOL002714	AR
Baicalein	MOL002714	DPP4
Baicalein	MOL002714	PIK3CG
Baicalein	MOL002714	NCOA2
Baicalein	MOL002714	NCOA1
Baicalein	MOL002714	RELA
Baicalein	MOL002714	AKT1
Baicalein	MOL002714	BCL2
Baicalein	MOL002714	BAX
Baicalein	MOL002714	MMP9
Baicalein	MOL002714	CASP3
Baicalein	MOL002714	TP53
Baicalein	MOL002714	HIF1A
Baicalein	MOL002714	FOSL1

Baicalein	MOL002714	FOSL2
Baicalein	MOL002714	CCNB1
Baicalein	MOL002714	MPO
Baicalein	MOL002714	AHR
Baicalein	MOL002714	IGF2
Baicalein	MOL002714	CYCS
Baicalein	MOL002714	NFATC1
Baicalein	MOL002714	TDRD7
Baicalein	MOL002714	EGLN1
Baicalein	MOL002714	NOX5
Baicalein	MOL002714	APOD
Luteolin	MOL000006	PTGS1
Luteolin	MOL000006	PTGS2
Luteolin	MOL000006	AR
Luteolin	MOL000006	NCOA2
Luteolin	MOL000006	DPP4
Luteolin	MOL000006	PIK3CG
Luteolin	MOL000006	RELA
Luteolin	MOL000006	EGFR
Luteolin	MOL000006	AKT1
Luteolin	MOL000006	CCND1
Luteolin	MOL000006	BCL2L1
Luteolin	MOL000006	CDKN1A
Luteolin	MOL000006	CASP9
Luteolin	MOL000006	MMP2
Luteolin	MOL000006	MMP9
Luteolin	MOL000006	MAPK1
Luteolin	MOL000006	IL10
Luteolin	MOL000006	RB1
Luteolin	MOL000006	TNF
Luteolin	MOL000006	IL6
Luteolin	MOL000006	CASP3
Luteolin	MOL000006	TP53
Luteolin	MOL000006	NFKBIA
Luteolin	MOL000006	XDH
Luteolin	MOL000006	TOP1
Luteolin	MOL000006	MDM2
Luteolin	MOL000006	MMP1
Luteolin	MOL000006	PCNA
Luteolin	MOL000006	ERBB2
Luteolin	MOL000006	PPARG
Luteolin	MOL000006	HMOX1
Luteolin	MOL000006	CASP7
Luteolin	MOL000006	ICAM1

Luteolin	MOL000006	MCL1
Luteolin	MOL000006	BIRC5
Luteolin	MOL000006	IL2
Luteolin	MOL000006	CCNB1
Luteolin	MOL000006	TYR
Luteolin	MOL000006	IFNG
Luteolin	MOL000006	IL4
Luteolin	MOL000006	TOP2A
Luteolin	MOL000006	GSTP1
Luteolin	MOL000006	SLC2A4
Luteolin	MOL000006	INSR
Luteolin	MOL000006	CD40LG
Luteolin	MOL000006	PTGES
Luteolin	MOL000006	NUF2
Luteolin	MOL000006	ADCY2
Luteolin	MOL000006	MET
Genkwanin	MOL005573	ADORA1
Genkwanin	MOL005573	ADORA2A
Genkwanin	MOL005573	ESR2
Genkwanin	MOL005573	PIM1
Genkwanin	MOL005573	ESR1
Genkwanin	MOL005573	ABCB1
Genkwanin	MOL005573	AKR1B1
Genkwanin	MOL005573	HSD17B1
Genkwanin	MOL005573	CYP1B1
Genkwanin	MOL005573	FLT3
Genkwanin	MOL005573	ABCG2
Genkwanin	MOL005573	PTGS2
Genkwanin	MOL005573	CDK5R1
Genkwanin	MOL005573	XDH
Genkwanin	MOL005573	CYP19A1
Genkwanin	MOL005573	CCNB3
Genkwanin	MOL005573	CDK6
Genkwanin	MOL005573	NOX4
Genkwanin	MOL005573	ABCC1
Genkwanin	MOL005573	PLG
Genkwanin	MOL005573	PTPRS
Genkwanin	MOL005573	AMY1A
Genkwanin	MOL005573	GRK6
Genkwanin	MOL005573	TNKS2
Genkwanin	MOL005573	TNKS
Genkwanin	MOL005573	MAOA
Genkwanin	MOL005573	ACHE
Genkwanin	MOL005573	SYK

Genkwanin	MOL005573	GSK3B
Genkwanin	MOL005573	TTR
Genkwanin	MOL005573	CSNK2A1
Genkwanin	MOL005573	CFTR
Genkwanin	MOL005573	AKR1B10
Genkwanin	MOL005573	CA2
Genkwanin	MOL005573	CA1
Genkwanin	MOL005573	CA12
Genkwanin	MOL005573	CA9
Genkwanin	MOL005573	AR
Genkwanin	MOL005573	CA7
Genkwanin	MOL005573	CA4
Genkwanin	MOL005573	CBR1
Genkwanin	MOL005573	TERT
Genkwanin	MOL005573	SLC22A12
Genkwanin	MOL005573	APP
Genkwanin	MOL005573	HSD17B2
Genkwanin	MOL005573	ADORA3
Genkwanin	MOL005573	KIT
Genkwanin	MOL005573	OPRD1
Genkwanin	MOL005573	EGFR
Genkwanin	MOL005573	KDM4E
Genkwanin	MOL005573	ALOX15
Genkwanin	MOL005573	CDK1
Genkwanin	MOL005573	ALOX12
Genkwanin	MOL005573	NOS2
Genkwanin	MOL005573	ALOX5
Genkwanin	MOL005573	MCL1
Genkwanin	MOL005573	NAE1
Genkwanin	MOL005573	PARP1
Genkwanin	MOL005573	LCK
Genkwanin	MOL005573	PLA2G2A
Genkwanin	MOL005573	SIGMAR1
Genkwanin	MOL005573	PFKFB3
Genkwanin	MOL005573	IGF1R
Genkwanin	MOL005573	ARG1
Genkwanin	MOL005573	GLO1
Genkwanin	MOL005573	MMP9
Genkwanin	MOL005573	MMP2
Genkwanin	MOL005573	MMP12
Genkwanin	MOL005573	CD38
Genkwanin	MOL005573	TOP1
Genkwanin	MOL005573	PIK3CG
Genkwanin	MOL005573	TYR

Genkwanin	MOL005573	AHR
Genkwanin	MOL005573	ESRRA
Genkwanin	MOL005573	ODC1
Genkwanin	MOL005573	ST6GAL1
Genkwanin	MOL005573	F2
Genkwanin	MOL005573	CALM1
Genkwanin	MOL005573	KDM5A
Genkwanin	MOL005573	PPARG
Genkwanin	MOL005573	CYP1A1
Genkwanin	MOL005573	CYP1A2
Genkwanin	MOL005573	PTPN1
Genkwanin	MOL005573	MET
Genkwanin	MOL005573	OPRM1
Genkwanin	MOL005573	IKBKB
Genkwanin	MOL005573	NTRK2
Genkwanin	MOL005573	BACE1
Genkwanin	MOL005573	AURKB
Genkwanin	MOL005573	SRC
Genkwanin	MOL005573	MAOB
Genkwanin	MOL005573	PDE4D
Genkwanin	MOL005573	GPR35
Genkwanin	MOL005573	DAPK1
Genkwanin	MOL005573	MPG
Genkwanin	MOL005573	BCHE
Genkwanin	MOL005573	FYN
Genkwanin	MOL005573	TACR2
Genkwanin	MOL005573	PRKDC
Genkwanin	MOL005573	MAPK3
Genkwanin	MOL005573	NOS2
Genkwanin	MOL005573	PTGS1
Genkwanin	MOL005573	PTGS2
Genkwanin	MOL005573	AR
Genkwanin	MOL005573	RXRA
Genkwanin	MOL005573	ESR2
Genkwanin	MOL005573	DPP4
Genkwanin	MOL005573	NCOA2
Genkwanin	MOL005573	NCOA1
Genkwanin	MOL005573	CHEK1
Baicalin	MOL002776	AKR1B1
Baicalin	MOL002776	ADORA1
Baicalin	MOL002776	TNF
Baicalin	MOL002776	IL2
Baicalin	MOL002776	XDH
Baicalin	MOL002776	RPS6KA3

Baicalin	MOL002776	EGFR
Baicalin	MOL002776	ACHE
Baicalin	MOL002776	NQO2
Baicalin	MOL002776	NMUR2
Baicalin	MOL002776	ADRA2A
Baicalin	MOL002776	ADRA2C
Baicalin	MOL002776	NOX4
Baicalin	MOL002776	ALDH2
Pectolinarigenin	MOL005842	KIT
Pectolinarigenin	MOL005842	OPRD1
Pectolinarigenin	MOL005842	AKR1B1
Pectolinarigenin	MOL005842	PIM1
Pectolinarigenin	MOL005842	ADORA1
Pectolinarigenin	MOL005842	ADORA2A
Pectolinarigenin	MOL005842	ADORA3
Pectolinarigenin	MOL005842	FLT3
Pectolinarigenin	MOL005842	CYP1B1
Pectolinarigenin	MOL005842	NOS2
Pectolinarigenin	MOL005842	NOX4
Pectolinarigenin	MOL005842	HSD17B1
Pectolinarigenin	MOL005842	ESR1
Pectolinarigenin	MOL005842	ESR2
Pectolinarigenin	MOL005842	ABCB1
Pectolinarigenin	MOL005842	ALOX15
Pectolinarigenin	MOL005842	ALOX12
Pectolinarigenin	MOL005842	ABCC1
Pectolinarigenin	MOL005842	APP
Pectolinarigenin	MOL005842	PTGS2
Pectolinarigenin	MOL005842	CFTR
Pectolinarigenin	MOL005842	GRK6
Pectolinarigenin	MOL005842	PTPRS
Pectolinarigenin	MOL005842	XDH
Pectolinarigenin	MOL005842	GSK3B
Pectolinarigenin	MOL005842	PLA2G2A
Pectolinarigenin	MOL005842	CA2
Pectolinarigenin	MOL005842	CA4
Pectolinarigenin	MOL005842	CDK1
Pectolinarigenin	MOL005842	CA9
Pectolinarigenin	MOL005842	HSD17B2
Pectolinarigenin	MOL005842	CYP19A1
Pectolinarigenin	MOL005842	MMP9
Pectolinarigenin	MOL005842	MMP2
Pectolinarigenin	MOL005842	OPRM1
Pectolinarigenin	MOL005842	CA7

Pectolinarigenin	MOL005842	CA12
Pectolinarigenin	MOL005842	CA1
Pectolinarigenin	MOL005842	CDK5R1
Pectolinarigenin	MOL005842	MAOA
Pectolinarigenin	MOL005842	NAE1
Pectolinarigenin	MOL005842	AMY1A
Pectolinarigenin	MOL005842	ODC1
Pectolinarigenin	MOL005842	CCNB3
Pectolinarigenin	MOL005842	CDK6
Pectolinarigenin	MOL005842	LCK
Pectolinarigenin	MOL005842	PIK3CG
Pectolinarigenin	MOL005842	ABCG2
Pectolinarigenin	MOL005842	ALOX5
Pectolinarigenin	MOL005842	KDM4E
Pectolinarigenin	MOL005842	CBR1
Pectolinarigenin	MOL005842	SIRT1
Pectolinarigenin	MOL005842	BACE1
Pectolinarigenin	MOL005842	TNKS2
Pectolinarigenin	MOL005842	TNKS
Pectolinarigenin	MOL005842	NTRK2
Pectolinarigenin	MOL005842	BCHE
Pectolinarigenin	MOL005842	CSNK2A1
Pectolinarigenin	MOL005842	ACHE
Pectolinarigenin	MOL005842	MCL1
Pectolinarigenin	MOL005842	TERT
Pectolinarigenin	MOL005842	EGFR
Pectolinarigenin	MOL005842	PLG
Pectolinarigenin	MOL005842	CALM1
Pectolinarigenin	MOL005842	MAOB
Pectolinarigenin	MOL005842	SYK
Pectolinarigenin	MOL005842	TTR
Pectolinarigenin	MOL005842	AKR1B10
Pectolinarigenin	MOL005842	ST6GAL1
Pectolinarigenin	MOL005842	CA6
Pectolinarigenin	MOL005842	AKT1
Pectolinarigenin	MOL005842	ALK
Pectolinarigenin	MOL005842	SLC22A12
Pectolinarigenin	MOL005842	SIGMAR1
Pectolinarigenin	MOL005842	AR
Pectolinarigenin	MOL005842	CYP1A1
Pectolinarigenin	MOL005842	CYP1A2
Pectolinarigenin	MOL005842	GLO1
Pectolinarigenin	MOL005842	ARG1
Pectolinarigenin	MOL005842	PFKFB3

Pectolinarigenin	MOL005842	IKBKB
Pectolinarigenin	MOL005842	MET
Pectolinarigenin	MOL005842	PARP1
Pectolinarigenin	MOL005842	MMP12
Pectolinarigenin	MOL005842	CD38
Pectolinarigenin	MOL005842	TOP1
Pectolinarigenin	MOL005842	HSP90B1
Pectolinarigenin	MOL005842	F2
Pectolinarigenin	MOL005842	AVPR2
Pectolinarigenin	MOL005842	CXCR1
Pectolinarigenin	MOL005842	GPR35
Pectolinarigenin	MOL005842	ALDH2
Pectolinarigenin	MOL005842	IGF1R
Pectolinarigenin	MOL005842	PPARG
Pectolinarigenin	MOL005842	PTPN1
Pectolinarigenin	MOL005842	TYR
Pectolinarigenin	MOL005842	AHR
Pectolinarigenin	MOL005842	ESRRA
Pectolinarigenin	MOL005842	HSP90AB1
Pectolinarigenin	MOL005842	AURKB
Pectolinarigenin	MOL005842	NOS2
Pectolinarigenin	MOL005842	SCN5A
Pectolinarigenin	MOL005842	PTGS2
Pectolinarigenin	MOL005842	ACHE
Pectolinarigenin	MOL005842	ADRA1B
Pectolinarigenin	MOL005842	DPP4
Pectolinarigenin	MOL005842	NCOA2
Pectolinarigenin	MOL005842	NCOA1
Pectolinarigenin	MOL005842	PTGS1
Oroxylin A	MOL002928	FLT3
Oroxylin A	MOL002928	NOS2
Oroxylin A	MOL002928	PTGS2
Oroxylin A	MOL002928	ABCB1
Oroxylin A	MOL002928	KIT
Oroxylin A	MOL002928	OPRD1
Oroxylin A	MOL002928	KDM4E
Oroxylin A	MOL002928	XDH
Oroxylin A	MOL002928	ALOX15
Oroxylin A	MOL002928	CDK1
Oroxylin A	MOL002928	ALOX12
Oroxylin A	MOL002928	GRK6
Oroxylin A	MOL002928	PIM1
Oroxylin A	MOL002928	ADORA1
Oroxylin A	MOL002928	ADORA2A

Oroxylin A	MOL002928	ESR2
Oroxylin A	MOL002928	AKR1B1
Oroxylin A	MOL002928	LCK
Oroxylin A	MOL002928	PTPRS
Oroxylin A	MOL002928	ESR1
Oroxylin A	MOL002928	ADORA3
Oroxylin A	MOL002928	CYP1B1
Oroxylin A	MOL002928	HSD17B1
Oroxylin A	MOL002928	MAOA
Oroxylin A	MOL002928	SYK
Oroxylin A	MOL002928	AKR1B10
Oroxylin A	MOL002928	ABCG2
Oroxylin A	MOL002928	AR
Oroxylin A	MOL002928	PLG
Oroxylin A	MOL002928	CDK5R1
Oroxylin A	MOL002928	CYP19A1
Oroxylin A	MOL002928	CA2
Oroxylin A	MOL002928	CCNB3
Oroxylin A	MOL002928	CA7
Oroxylin A	MOL002928	CDK6
Oroxylin A	MOL002928	CA1
Oroxylin A	MOL002928	CA12
Oroxylin A	MOL002928	CA9
Oroxylin A	MOL002928	CA4
Oroxylin A	MOL002928	CBR1
Oroxylin A	MOL002928	IKBKB
Oroxylin A	MOL002928	NTRK2
Oroxylin A	MOL002928	ODC1
Oroxylin A	MOL002928	MMP12
Oroxylin A	MOL002928	CD38
Oroxylin A	MOL002928	TOP1
Oroxylin A	MOL002928	ARG1
Oroxylin A	MOL002928	MMP9
Oroxylin A	MOL002928	MMP2
Oroxylin A	MOL002928	PIK3CG
Oroxylin A	MOL002928	GSK3B
Oroxylin A	MOL002928	ABCC1
Oroxylin A	MOL002928	CALM1
Oroxylin A	MOL002928	PLA2G2A
Oroxylin A	MOL002928	ACHE
Oroxylin A	MOL002928	SLC22A12
Oroxylin A	MOL002928	TERT
Oroxylin A	MOL002928	AMY1A
Oroxylin A	MOL002928	TNKS

Oroxylin A	MOL002928	TTR
Oroxylin A	MOL002928	HSD17B2
Oroxylin A	MOL002928	TYR
Oroxylin A	MOL002928	AHR
Oroxylin A	MOL002928	ESRRA
Oroxylin A	MOL002928	PDE5A
Oroxylin A	MOL002928	APP
Oroxylin A	MOL002928	MCL1
Oroxylin A	MOL002928	NAE1
Oroxylin A	MOL002928	EGFR
Oroxylin A	MOL002928	SIGMAR1
Oroxylin A	MOL002928	TNKS2
Oroxylin A	MOL002928	NOX4
Oroxylin A	MOL002928	CYP1A1
Oroxylin A	MOL002928	OPRM1
Oroxylin A	MOL002928	CXCR1
Oroxylin A	MOL002928	CSNK2A1
Oroxylin A	MOL002928	MAPT
Oroxylin A	MOL002928	TOP2A
Oroxylin A	MOL002928	INSR
Oroxylin A	MOL002928	MYLK
Oroxylin A	MOL002928	MPO
Oroxylin A	MOL002928	PIK3R1
Oroxylin A	MOL002928	DAPK1
Oroxylin A	MOL002928	PYGL
Oroxylin A	MOL002928	MMP13
Oroxylin A	MOL002928	MMP3
Oroxylin A	MOL002928	CA3
Oroxylin A	MOL002928	CA14
Oroxylin A	MOL002928	CA13
Oroxylin A	MOL002928	PLA2G1B
Oroxylin A	MOL002928	CA5A
Oroxylin A	MOL002928	APEX1
Oroxylin A	MOL002928	AKR1C2
Oroxylin A	MOL002928	AKR1C1
Oroxylin A	MOL002928	AKR1C3
Oroxylin A	MOL002928	AKR1C4
Oroxylin A	MOL002928	AKR1A1
Oroxylin A	MOL002928	MPG
Oroxylin A	MOL002928	GPR35
Oroxylin A	MOL002928	BCHE
Oroxylin A	MOL002928	NOS2
Oroxylin A	MOL002928	PTGS1
Oroxylin A	MOL002928	PTGS2

Oroxylin A	MOL002928	AR
Oroxylin A	MOL002928	SCN5A
Oroxylin A	MOL002928	RXRA
Oroxylin A	MOL002928	ADRA1B
Oroxylin A	MOL002928	ADRB2
Oroxylin A	MOL002928	DPP4
Oroxylin A	MOL002928	PIK3CG
Oroxylin A	MOL002928	NCOA1
Oroxylin A	MOL002928	NCOA2
Oroxylin A	MOL002928	PKIA
Oroxylin A	MOL002928	BCL2
Oroxylin A	MOL002928	IL6
Oroxylin A	MOL002928	CASP3
Oroxylin A	MOL002928	CYP1A2
Oroxylin A	MOL002928	CCNB1
Oroxylin A	MOL002928	CYP2C9
Kaempferol	MOL000422	NMUR2
Kaempferol	MOL000422	ADRA2A
Kaempferol	MOL000422	ADRA2C
Kaempferol	MOL000422	ACHE
Kaempferol	MOL000422	AKR1B1
Kaempferol	MOL000422	CA7
Kaempferol	MOL000422	CA12
Kaempferol	MOL000422	CA4
Kaempferol	MOL000422	CA2
Kaempferol	MOL000422	NOX4
Kaempferol	MOL000422	NQO2
Kaempferol	MOL000422	RPS6KA3
Kaempferol	MOL000422	XDH
Kaempferol	MOL000422	CD38
Kaempferol	MOL000422	PTGS2
Kaempferol	MOL000422	PDE5A
Kaempferol	MOL000422	ADORA1
Kaempferol	MOL000422	TNF
Kaempferol	MOL000422	IL2
Kaempferol	MOL000422	ALOX5
Morin	MOL000737	PTPRS
Morin	MOL000737	ESR2
Morin	MOL000737	DAPK1
Morin	MOL000737	MPG
Morin	MOL000737	SLC22A12
Morin	MOL000737	NOX4
Morin	MOL000737	XDH
Morin	MOL000737	TYR

Morin	MOL000737	FLT3
Morin	MOL000737	ALOX5
Morin	MOL000737	HSD17B2
Morin	MOL000737	ABCC1
Morin	MOL000737	HSD17B1
Morin	MOL000737	AHR
Morin	MOL000737	ESRRA
Morin	MOL000737	ABCB1
Morin	MOL000737	CYP1B1
Morin	MOL000737	ABCG2
Morin	MOL000737	BCHE
Morin	MOL000737	ACHE
Morin	MOL000737	ADORA1
Morin	MOL000737	ADORA2A
Morin	MOL000737	ADORA3
Morin	MOL000737	GPR35
Morin	MOL000737	ALOX15
Morin	MOL000737	MAPT
Morin	MOL000737	KDM4E
Morin	MOL000737	AVPR2
Morin	MOL000737	TOP2A
Morin	MOL000737	MAOA
Morin	MOL000737	CYP19A1
Morin	MOL000737	PIM1
Morin	MOL000737	DRD4
Morin	MOL000737	GLO1
Morin	MOL000737	MYLK
Morin	MOL000737	MPO
Morin	MOL000737	PIK3R1
Morin	MOL000737	PYGL
Morin	MOL000737	SYK
Morin	MOL000737	MMP13
Morin	MOL000737	MMP3
Morin	MOL000737	CA3
Morin	MOL000737	CDK1
Morin	MOL000737	MMP9
Morin	MOL000737	PIK3CG
Morin	MOL000737	MMP2
Morin	MOL000737	PKN1
Morin	MOL000737	NEK2
Morin	MOL000737	CXCR1
Morin	MOL000737	CAMK2B
Morin	MOL000737	ALK
Morin	MOL000737	NEK6

Morin	MOL000737	PLA2G1B
Morin	MOL000737	AXL
Morin	MOL000737	APEX1
Morin	MOL000737	AKR1C2
Morin	MOL000737	AKR1C1
Morin	MOL000737	AKR1C3
Morin	MOL000737	AKR1C4
Morin	MOL000737	AKR1A1
Morin	MOL000737	AKR1B1
Morin	MOL000737	CA2
Morin	MOL000737	CA7
Morin	MOL000737	CA12
Morin	MOL000737	PFKFB3
Morin	MOL000737	CDK5R1
Morin	MOL000737	CCNB3
Morin	MOL000737	CDK6
Morin	MOL000737	CDK2
Morin	MOL000737	ARG1
Morin	MOL000737	NAE1
Morin	MOL000737	ESR1
Morin	MOL000737	PTGS2
Morin	MOL000737	TTR
Morin	MOL000737	CFTR
Morin	MOL000737	AKR1B10
Morin	MOL000737	TNKS2
Morin	MOL000737	TNKS
Morin	MOL000737	CA4
Morin	MOL000737	MCL1
Morin	MOL000737	AMY1A
Morin	MOL000737	GRK6
Morin	MOL000737	F2
Morin	MOL000737	ALOX12
Morin	MOL000737	AR
Morin	MOL000737	CBR1
Morin	MOL000737	TERT
Morin	MOL000737	IGF1R
Morin	MOL000737	INSR
Morin	MOL000737	EGFR
Morin	MOL000737	AURKB
Morin	MOL000737	CA1
Morin	MOL000737	GSK3B
Morin	MOL000737	SRC
Morin	MOL000737	PTK2
Morin	MOL000737	KDR

Morin	MOL000737	PLK1
Morin	MOL000737	CA6
Morin	MOL000737	CA14
Morin	MOL000737	CA9
Morin	MOL000737	PTGS1
Morin	MOL000737	PTGS2
Morin	MOL000737	AR
Morin	MOL000737	PPARG
Morin	MOL000737	DPP4
Morin	MOL000737	PIK3CG
Morin	MOL000737	XDH
Morin	MOL000737	TOP1
Morin	MOL000737	EDN1
Morin	MOL000737	CD36
Morin	MOL000737	DIO1
Morin	MOL000737	GSR
Morin	MOL000737	PIP4K2A
Morin	MOL000737	BATF3
Praeruptorin A	MOL013079	TNNC1
Praeruptorin A	MOL013079	FLT1
Praeruptorin A	MOL013079	KDR
Praeruptorin A	MOL013079	GRM5
Praeruptorin A	MOL013079	SCN9A
Praeruptorin A	MOL013079	CDK9
Praeruptorin A	MOL013079	NOS1
Praeruptorin A	MOL013079	NOS2
Praeruptorin A	MOL013079	NOS3
Praeruptorin A	MOL013079	CDC7
Praeruptorin A	MOL013079	HTR2B
Praeruptorin A	MOL013079	HCRTR2
Praeruptorin A	MOL013079	HCRTR1
Praeruptorin A	MOL013079	IGF1R
Praeruptorin A	MOL013079	OPRK1
Praeruptorin A	MOL013079	TSPO
Praeruptorin A	MOL013079	PARP1
Praeruptorin A	MOL013079	PARP3
Praeruptorin A	MOL013079	GRM2
Praeruptorin A	MOL013079	CYP19A1
Praeruptorin A	MOL013079	GABRB3
Praeruptorin A	MOL013079	ALOX5
Praeruptorin A	MOL013079	KCNK3
Praeruptorin A	MOL013079	KCNK9
Praeruptorin A	MOL013079	GSK3B
Praeruptorin A	MOL013079	GRM3

Praeruptorin A	MOL013079	PTGS2
Praeruptorin A	MOL013079	CDK2
Praeruptorin A	MOL013079	CDK4
Praeruptorin A	MOL013079	JAK3
Praeruptorin A	MOL013079	JAK2
Praeruptorin A	MOL013079	CPT1A
Praeruptorin A	MOL013079	DAPK3
Praeruptorin A	MOL013079	ALK
Praeruptorin A	MOL013079	PDE10A
Praeruptorin A	MOL013079	CASP3
Praeruptorin A	MOL013079	CASP7
Praeruptorin A	MOL013079	NPY5R
Praeruptorin A	MOL013079	IDH1
Praeruptorin A	MOL013079	CCNE2
Praeruptorin A	MOL013079	CCNB3
Praeruptorin A	MOL013079	CDK2
Praeruptorin A	MOL013079	CCND3
Praeruptorin A	MOL013079	CHRM2
Praeruptorin A	MOL013079	CHRM1
Praeruptorin A	MOL013079	DRD4
Praeruptorin A	MOL013079	CHUK
Praeruptorin A	MOL013079	ADAM17
Praeruptorin A	MOL013079	LRRK2
Praeruptorin A	MOL013079	SCN5A
Praeruptorin A	MOL013079	CYP1A1
Praeruptorin A	MOL013079	ADORA1
Praeruptorin A	MOL013079	MAPK8
Praeruptorin A	MOL013079	ADORA3
Praeruptorin A	MOL013079	MAPK10
Praeruptorin A	MOL013079	PGK1
Praeruptorin A	MOL013079	PLK1
Praeruptorin A	MOL013079	MALT1
Praeruptorin A	MOL013079	CMA1
Praeruptorin A	MOL013079	MAPK9
Praeruptorin A	MOL013079	NAMPT
Praeruptorin A	MOL013079	NR3C1
Praeruptorin A	MOL013079	PTK2
Praeruptorin A	MOL013079	CCKBR
Praeruptorin A	MOL013079	ROCK1
Praeruptorin A	MOL013079	CRHR1
Praeruptorin A	MOL013079	EGFR
Praeruptorin A	MOL013079	CNR1
Praeruptorin A	MOL013079	GRM4
Praeruptorin A	MOL013079	IRAK4

Praeruptorin A	MOL013079	CSF1R
Praeruptorin A	MOL013079	CHRM5
Praeruptorin A	MOL013079	ITGB7
Praeruptorin A	MOL013079	ADORA2A
Praeruptorin A	MOL013079	CTSK
Praeruptorin A	MOL013079	PREP
Praeruptorin A	MOL013079	FAP
Praeruptorin A	MOL013079	CXCR2
Praeruptorin A	MOL013079	ROCK2
Praeruptorin A	MOL013079	LIMK1
Praeruptorin A	MOL013079	MAPK1
Praeruptorin A	MOL013079	PRKACA
Praeruptorin A	MOL013079	CYP26A1
Praeruptorin A	MOL013079	LIMK2
Praeruptorin A	MOL013079	SYK
Praeruptorin A	MOL013079	CSNK1D
Praeruptorin A	MOL013079	POLR1A
Praeruptorin A	MOL013079	P2RX7
Praeruptorin A	MOL013079	CSNK1E
Praeruptorin A	MOL013079	STK33
Praeruptorin A	MOL013079	CCNE1
Praeruptorin A	MOL013079	PDGFRB
Praeruptorin A	MOL013079	KIT
Praeruptorin A	MOL013079	MAPKAPK2
Praeruptorin A	MOL013079	NTRK1
Praeruptorin A	MOL013079	BDKRB1
Praeruptorin A	MOL013079	PTGES
Praeruptorin A	MOL013079	INSR
Praeruptorin A	MOL013079	ADORA2B
Praeruptorin A	MOL013079	PIK3CB
Praeruptorin A	MOL013079	KCNMA1
Praeruptorin A	MOL013079	CA2
Praeruptorin A	MOL013079	DPP4
Praeruptorin A	MOL013079	PTGS2
Praeruptorin A	MOL013079	RELA
Praeruptorin A	MOL013079	TNF
Marmesin	MOL001944	PTGS1
Marmesin	MOL001944	PTGS2
Marmesin	MOL001944	ESR1
Marmesin	MOL001944	AR
Marmesin	MOL001944	ADRB2
Marmesin	MOL001944	SLC6A4
Marmesin	MOL001944	DPP4
Marmesin	MOL001944	CHRM1

Marmesin	MOL001944	RXRA
Marmesin	MOL001944	CHRM2
Marmesin	MOL001944	PIK3CG
Taxifolin	MOL004576	PTGS1
Taxifolin	MOL004576	PTGS2
Taxifolin	MOL004576	PIK3CG
Taxifolin	MOL004576	RXRA
Taxifolin	MOL004576	RELA
Taxifolin	MOL004576	ICAM1
Taxifolin	MOL004576	DGAT2
Taxifolin	MOL004576	MTTP
Taxifolin	MOL004576	APOB
Licochalcone B	MOL004841	MAOB
Licochalcone B	MOL004841	CHRNA7
Licochalcone B	MOL004841	AKR1B1
Licochalcone B	MOL004841	BACE1
Licochalcone B	MOL004841	PTGS2
Licochalcone B	MOL004841	ALOX5
Licochalcone B	MOL004841	EGFR
Licochalcone B	MOL004841	F3
Licochalcone B	MOL004841	APP
Licochalcone B	MOL004841	ABCG2
Licochalcone B	MOL004841	CYP19A1
Licochalcone B	MOL004841	TERT
Licochalcone B	MOL004841	PTPN1
Licochalcone B	MOL004841	NOS2
Licochalcone B	MOL004841	SNCA
Licochalcone B	MOL004841	MAOA
Licochalcone B	MOL004841	ABCB1
Licochalcone B	MOL004841	CXCR4
Licochalcone B	MOL004841	TYR
Licochalcone B	MOL004841	ABCC1
Licochalcone B	MOL004841	SHBG
Licochalcone B	MOL004841	CBR1
Licochalcone B	MOL004841	HSD17B14
Licochalcone B	MOL004841	MMP2
Licochalcone B	MOL004841	ERN1
Licochalcone B	MOL004841	INSR
Licochalcone B	MOL004841	CLK1
Licochalcone B	MOL004841	RPS6KB1
Licochalcone B	MOL004841	TUBB1
Licochalcone B	MOL004841	IGF1R
Licochalcone B	MOL004841	PDK1
Licochalcone B	MOL004841	ODC1

Licochalcone B	MOL004841	GLI2
Licochalcone B	MOL004841	GLI1
Licochalcone B	MOL004841	BCL2L1
Licochalcone B	MOL004841	BCL2
Licochalcone B	MOL004841	CAPN1
Licochalcone B	MOL004841	WEE1
Licochalcone B	MOL004841	ESR1
Licochalcone B	MOL004841	ESR2
Licochalcone B	MOL004841	VCP
Licochalcone B	MOL004841	TYMS
Licochalcone B	MOL004841	NOX4
Licochalcone B	MOL004841	FLT3
Licochalcone B	MOL004841	PLK1
Licochalcone B	MOL004841	ESRRA
Licochalcone B	MOL004841	ESRRB
Licochalcone B	MOL004841	ABL1
Licochalcone B	MOL004841	PDGFRB
Licochalcone B	MOL004841	CDK4
Licochalcone B	MOL004841	CHEK1
Licochalcone B	MOL004841	ALPL
Licochalcone B	MOL004841	DUSP3
Licochalcone B	MOL004841	HDAC1
Licochalcone B	MOL004841	MAP4K4
Licochalcone B	MOL004841	MAPT
Licochalcone B	MOL004841	ADCY5
Licochalcone B	MOL004841	ALOX5AP
Licochalcone B	MOL004841	HPGDS
Licochalcone B	MOL004841	YWHAG
Licochalcone B	MOL004841	PGD
Licochalcone B	MOL004841	CYP1B1
Licochalcone B	MOL004841	CDK1
Licochalcone B	MOL004841	NQO2
Licochalcone B	MOL004841	ROCK1
Licochalcone B	MOL004841	HSD11B1
Licochalcone B	MOL004841	PLAU
Licochalcone B	MOL004841	RAF1
Licochalcone B	MOL004841	CA3
Licochalcone B	MOL004841	PIK3CB
Licochalcone B	MOL004841	PIK3CA
Licochalcone B	MOL004841	DNM1
Licochalcone B	MOL004841	BRAF
Licochalcone B	MOL004841	PTGS1
Licochalcone B	MOL004841	PDPK1
Licochalcone B	MOL004841	HDAC8

Licochalcone B	MOL004841	DRD5
Licochalcone B	MOL004841	DRD4
Licochalcone B	MOL004841	SIGMAR1
Licochalcone B	MOL004841	CHEK2
Licochalcone B	MOL004841	ALDH2
Licochalcone B	MOL004841	MTOR
Licochalcone B	MOL004841	IGFBP3
Licochalcone B	MOL004841	HDAC6
Licochalcone B	MOL004841	SRC
Licochalcone B	MOL004841	MMP12
Licochalcone B	MOL004841	NOS2
Licochalcone B	MOL004841	PTGS1
Licochalcone B	MOL004841	PTGS2
Licochalcone B	MOL004841	ESR1
Licochalcone B	MOL004841	AR
Licochalcone B	MOL004841	PPARG
Licochalcone B	MOL004841	CA2
Licochalcone B	MOL004841	ADRB2
Licochalcone B	MOL004841	ESR2
Licochalcone B	MOL004841	MAPK14
Licochalcone B	MOL004841	GSK3B
Licochalcone B	MOL004841	CHEK1
Licochalcone B	MOL004841	CCNA2
Brazilin	73384	EGFR
Brazilin	73384	ERBB2
Brazilin	73384	MET
Brazilin	73384	MAPT
Brazilin	73384	KIT
Brazilin	73384	FGFR1
Brazilin	73384	SRC
Brazilin	73384	FLT1
Brazilin	73384	KDR
Brazilin	73384	TDP1
Brazilin	73384	ERBB3
Brazilin	73384	ERBB4
Brazilin	73384	CSF1R
Brazilin	73384	FGFR2
Brazilin	73384	FGFR4
Cardamoni	641785	KCNA3
Cardamoni	641785	KCNA2
Cardamoni	641785	KCNA6
Cardamoni	641785	KCNA4
Cardamoni	641785	KCNA5
Cardamoni	641785	KCNA1

Cardamoni	641785	KCNA10
Cardamoni	641785	KCNA7
Cardamoni	641785	MAPT
Cardamoni	641785	ABCG2
Cardamoni	641785	PDPK1
Cardamoni	641785	AKR1B10
Cardamoni	641785	AKR1B1
Cardamoni	641785	CRYZ
Cardamoni	641785	AKR1B15
Iristectorigenin B	5488781	EGFR
Iristectorigenin B	5488781	ERBB2
Iristectorigenin B	5488781	ERBB3
Iristectorigenin B	5488781	ERBB4
Iristectorigenin B	5488781	MAPT
Iristectorigenin B	5488781	TDP1
Iristectorigenin B	5488781	CYP19A1
Iristectorigenin B	5488781	CBR1
Iristectorigenin B	5488781	HSD17B2
Iristectorigenin B	5488781	CBR3
Iristectorigenin B	5488781	HSD11B2
Iristectorigenin B	5488781	MIF
Iristectorigenin B	5488781	TYR
Iristectorigenin B	5488781	P05091
7,8-Dihydroxycoumarin	5280569	EGFR
7,9-Dihydroxycoumarin	5280569	ERBB2
7,10-Dihydroxycoumarin	5280569	ERBB3
7,11-Dihydroxycoumarin	5280569	ERBB4
7,12-Dihydroxycoumarin	5280569	CA12
7,13-Dihydroxycoumarin	5280569	CA9
7,14-Dihydroxycoumarin	5280569	CA14
7,15-Dihydroxycoumarin	5280569	XDH
7,16-Dihydroxycoumarin	5280569	AOX1
7,17-Dihydroxycoumarin	5280569	PLK4
7,18-Dihydroxycoumarin	5280569	AURKA
7,19-Dihydroxycoumarin	5280569	NUAK1
7,20-Dihydroxycoumarin	5280569	CA1
7,21-Dihydroxycoumarin	5280569	CA2
7,22-Dihydroxycoumarin	5280569	INSR
5-Hydroxy-6,7-dimethoxylflavone	471722	AKR1B1
5-Hydroxy-6,8-dimethoxylflavone	471722	ADORA2A
5-Hydroxy-6,9-dimethoxylflavone	471722	ADORA1
5-Hydroxy-6,10-dimethoxylflavone	471722	ADORA3
5-Hydroxy-6,11-dimethoxylflavone	471722	AKR1B15
5-Hydroxy-6,12-dimethoxylflavone	471722	AKR1B10

5-Hydroxy-6,13-dimethoxylflavone	471722	CREB1
5-Hydroxy-6,14-dimethoxylflavone	471722	ATF1
5-Hydroxy-6,15-dimethoxylflavone	471722	CREM
5-Hydroxy-6,16-dimethoxylflavone	471722	GFER
5-Hydroxy-6,17-dimethoxylflavone	471722	MAPT
5-Hydroxy-6,18-dimethoxylflavone	471722	ABCG2
5-Hydroxy-6,19-dimethoxylflavone	471722	KDM4E
5-Hydroxy-6,20-dimethoxylflavone	471722	KDM4A
5-Hydroxy-6,21-dimethoxylflavone	471722	CDK1
Isosakuranetin	160481	CYP1A2
Isosakuranetin	160481	CYP1B1
Isosakuranetin	160481	CYP1A1
Isosakuranetin	160481	MAPT
Isosakuranetin	160481	ADORA3
Isosakuranetin	160481	TAS2R31
Isosakuranetin	160481	ADORA1
Isosakuranetin	160481	TAS2R43
Isosakuranetin	160481	TAS2R46
Isosakuranetin	160481	TAS2R19
Isosakuranetin	160481	TAS2R20
Isosakuranetin	160481	TAS2R50
Isosakuranetin	160481	TAS2R14
Isosakuranetin	160481	TAS2R13
Isosakuranetin	160481	TAS2R10
Skimmin	99693	CA12
Skimmin	99693	CA1
Skimmin	99693	CA2
Skimmin	99693	CA3
Skimmin	99693	P35218
Skimmin	99693	CA7
Skimmin	99693	CA9
Skimmin	99693	CA13
Skimmin	99693	CA14
Skimmin	99693	Q9Y2D0
Skimmin	99693	DYRK1A
Skimmin	99693	AKR1B1
Skimmin	99693	AKR1B15
Skimmin	99693	AKR1B10
Skimmin	99693	P05091
